# CsTCPs regulate shoot tip development and catechin biosynthesis in tea plant (*Camellia sinensis*)

**DOI:** 10.1038/s41438-021-00538-7

**Published:** 2021-05-01

**Authors:** Shuwei Yu, Penghui Li, Xuecheng Zhao, Mangmang Tan, Muhammad Zulfiqar Ahmad, Yujie Xu, Million Tadege, Jian Zhao

**Affiliations:** 1 State Key Laboratory of Tea Plant Biology and Utilization, Anhui Agricultural University, Hefei, 230036 China; 2 Department of Plant and Soil Sciences, Institute for Agricultural Biosciences, Oklahoma State University, 3210 Sam Noble Parkway, Ardmore, OK 73401 USA

**Keywords:** Non-model organisms, Leaf development

## Abstract

The growth of leaves and biosynthesis of characteristic secondary metabolites are critically important for tea production and quality control. However, little is known about the coordinated regulation of leaf development and catechin biosynthesis in tea plants. Here, we reported that TCP TFs are involved in both catechin biosynthesis and leaf development. An integrated analysis of catechin profiling and *CsTCP* expression in different tissues of plants under various environmental conditions at different developmental stages indicated significant correlations between the transcript levels of CIN-type *TCPs* and catechin production. CIN-type CsTCP3 and CsTCP4 and PCF-type CsTCP14 interacted with the MYB-bHLH-WD40 repeat (MBW) complex by forming a CsTCP3-CsTT8 heterodimer and modulating the transactivation activity of the promoters of anthocyanin synthase (*CsANS1*) and anthocyanidin reductase (*CsANR1*). Four types of microRNA/target modules, *miR319b/CsTCP3-4*, *miR164b/CsCUC*, *miR396/CsGRF-GIF*, and *miR165b/HD-ZIPIII* ones, were also identified and characterized for their functions in the regulation of the development of tea plant shoot tips and leaf shape. The results of these modules were reflected by their different expression patterns in developing buds and leaves that had distinctly different morphologies in three different tea plant varieties. Their roles in the regulation of catechin biosynthesis were also further verified by manipulation of microRNA319b (*miR319b*), which targets the transcripts of *CsTCP3* and *CsTCP4*. Thus, CsTCPs represent at least one of these important groups of TFs that can integrate tea plant leaf development together with secondary metabolite biosynthesis. Our study provides new insight into shoot tip development and catechin production in tea plants and lays a foundation for further mechanistic understanding of the regulation of tea plant leaf development and secondary metabolism.

## Introduction

Tea plant [*Camellia sinensis* (L.) O. Kuntze] is a perennial evergreen tree or shrub species cultivated worldwide. Its tender shoot tips, including apical buds, young leaves, and stems are used for making various types of teas due to the concentrated presence of secondary metabolites such as catechins, caffeine, theanine, and terpenoid volatiles^1^. These characteristic secondary metabolites largely determine the pleasant flavors and numerous health benefits of tea beverages. Therefore, the production of these tender shoot tips with appropriate contents and composition of secondary metabolites is extremely important for tea production. While many studies in recent decades have mainly focused on plant secondary metabolites in tea plant leaves with regard to the biochemical pathways and molecular mechanisms underlying their biosynthesis and regulation and their health benefits^1,2^, the growth and production of tender shoot tips is not well understood, including shoot apical meristem (SAM) development, leaf initiation and development, internode elongation, trichome and stoma formation, branching, and the integrated control of secondary metabolite production during shoot tip development^1^. Several MYB transcription factors (TFs), such as CsAN1, CsTT2, and CsMYB75, have been reported to regulate anthocyanin and catechin biosynthesis^3–5^. The TFs that regulate the growth and development of shoot tips and the formation of tissues and organs in tea plants are largely unknown. Aboveground plant growth and development are obviously orchestrated by the SAM; leaves and branches arise from apical and axillary meristems, respectively, the process of which is controlled by multiple complex regulatory networks^6,7^. Such networks are also believed to operate in the shoot tips of tea plants, although the molecular coordination of this complex developmental process in terms of secondary metabolite production is poorly understood. Phytohormones such as auxin, cytokinin (CK), jasmonic acid (JA), and gibberellic acid (GA) and environmental factors can affect and alter shoot tip development and secondary metabolite production^6–8^ and are likely to be the key signaling integrators of growth and secondary metabolite accumulation in tea plants.

While a wide array of TFs may coordinate the development and biosynthesis of specialized metabolites in shoot tips, the members of the TEOSINTE BRANCHED 1 (TB1)/CYCLOIDEA (CYC)/PROLIFERATING CELL NUCLEAR ANTIGEN FACTOR 1 (PCF1) (TCP) family are particularly important^8,9^. TCPs are important plant growth regulators involved in cell proliferation in various organs and tissues, hormone synthesis and signaling, plant responses to abiotic and biotic stresses, and biosynthesis of plant secondary metabolites^10–12^. The plant TCP TF family comprises two distinct classes: class-I TCP PCFs and class-II TCPs, which in turn can be divided into CIN-TCP and CYC-TCP subclades. Class-I TCPs regulate plant cell proliferation, leaf development, and stem elongation and modulate hormone biosynthesis and signaling^13,14^. Class-I TCP TFs redundantly promote plant growth, as reflected by OsPCF1/2 and AtTCP20 activation of *CYCB1;1* genes^6,7,13–15^. On the other hand, class-II TCP TFs often inhibit growth and proliferation. CIN-TCPs are involved in lateral organ development, and CYC/TB1 TCP TFs regulate the development of the axillary meristem (AM) into either flower petals or lateral shoots^6,7,16–18^. CIN-TCP TFs repress cell proliferation in developing leaf primordia, and compared with the wild type, mutants defective in the genes responsible for this phenomena displayed longer periods of cell division and larger leaves with altered shapes and/or crinkled surfaces^19–22^. CYC-TCPs, such as Arabidopsis BRANCHED 1 (AtBRC1)^23^ and rice TEOSINTE BRANCHED 1 (OsTB1)^24^, act as repressors of axillary bud outgrowth and branching. The Arabidopsis class-I TCPs AtTCP14 and AtTCP15 redundantly function in the regulation of plant development, including seed germination^25^, leaf shape^26^, inflorescence stem growth^27^, and inhibition of anthocyanin synthesis during exposure to high-light intensity, by modulating *PRODUCTION OF ANTHOCYANIN PIGMENT 1* (*PAP1*) and *TRANPARENT TESTA 8* (*TT8*) expression^28^. By contrast, the class-II AtTCP3 acts as an activator of anthocyanin biosynthesis-related genes by altering the Myb-bHLH-WD40 (MBW) complex^29^, indicating that the function of class-II TCPs is usually opposite that of class-I TCPs in regulating plant growth and development, organogenesis, and hormone responses^6,7^.

MicroRNAs (miRNAs), together with their target genes, essentially regulate leaf differentiation and growth^19–22^. These miRNAs include *miR319* and its target *CIN-TCP*s (abbreviated as *miR319*/*CIN-TCP*s), *miR164/CUP-SHAPED COTYLEDON 1* (*CUC1*), *miR165*/*class-III homeodomain-leucine zipper* (*HD-ZIPIII*), *miR396*/*GROWTH REGULATING* FACTORs (*GRF*s), and *GRF-INTERACTING* FACTORs (*GIF*s)^19–22,30–32^. Mutants with altered miRNA metabolism have pleiotropic developmental defects^19,20,30–32^. For example, the JAW locus, which encodes microRNA319 (*miRNA319*) and represses expression of the *AtTCP3*, *AtTCP4*, *AtTCP10* genes, controls leaf development^6,19^. The *miRNA319*-guided cleavage of *AtTCP4* and its homologs is necessary to prevent aberrant activity of *AtTCP4* due to improper gene expression^19^. Overexpression of wild-type and miRNA-resistant *AtTCP4* demonstrated that *miRNA319* regulation is sufficient to restrict *AtTCP4* function to its normal activity^19^, and such regulatory module-mediated leaf morphogenesis is usually conserved^33,34^. The concurrence of characteristic secondary metabolite biosynthesis with the growth and development of apical buds and young leaves suggests that these processes may be coordinately regulated in tea plants, although the underlying regulatory mechanisms remain unknown. It is highly important to understand these associations, since both the growth of shoot tips and accumulation of valuable secondary metabolites are major traits in terms of tea production and the nutritional quality of tea. Given the various and essential roles of TCP TFs in the regulation of the development of leaf shape, stem branching, and secondary metabolite biosynthesis, we conducted an expression analysis of tea plant *CsTCP* family genes and functionally characterized them. Their expression profiles in comparison with those of functionally known homologs in Arabidopsis and correlation analyses integrating transcriptome and catechin profiling of shoot tips at various developmental stages and under various environmental and hormone stresses enabled us to dissect the putative functions of *CsTCP* genes. The functions of miRNAs together with their target TFs, such as *CsmiR319/CsTCP3* and *CsTCP4*, in the regulation of leaf development and catechin biosynthesis were determined. Our study provides new insight into the coordinated regulation of shoot tip development and secondary metabolite accumulation and lays a foundation for the future elucidation of the mechanisms underlying tea plant leaf development and the development of markers for breeding better tea plant varieties.

## Results

### Identification of 35 *TCP* genes in the tea plant genome

**Fig. 1:**
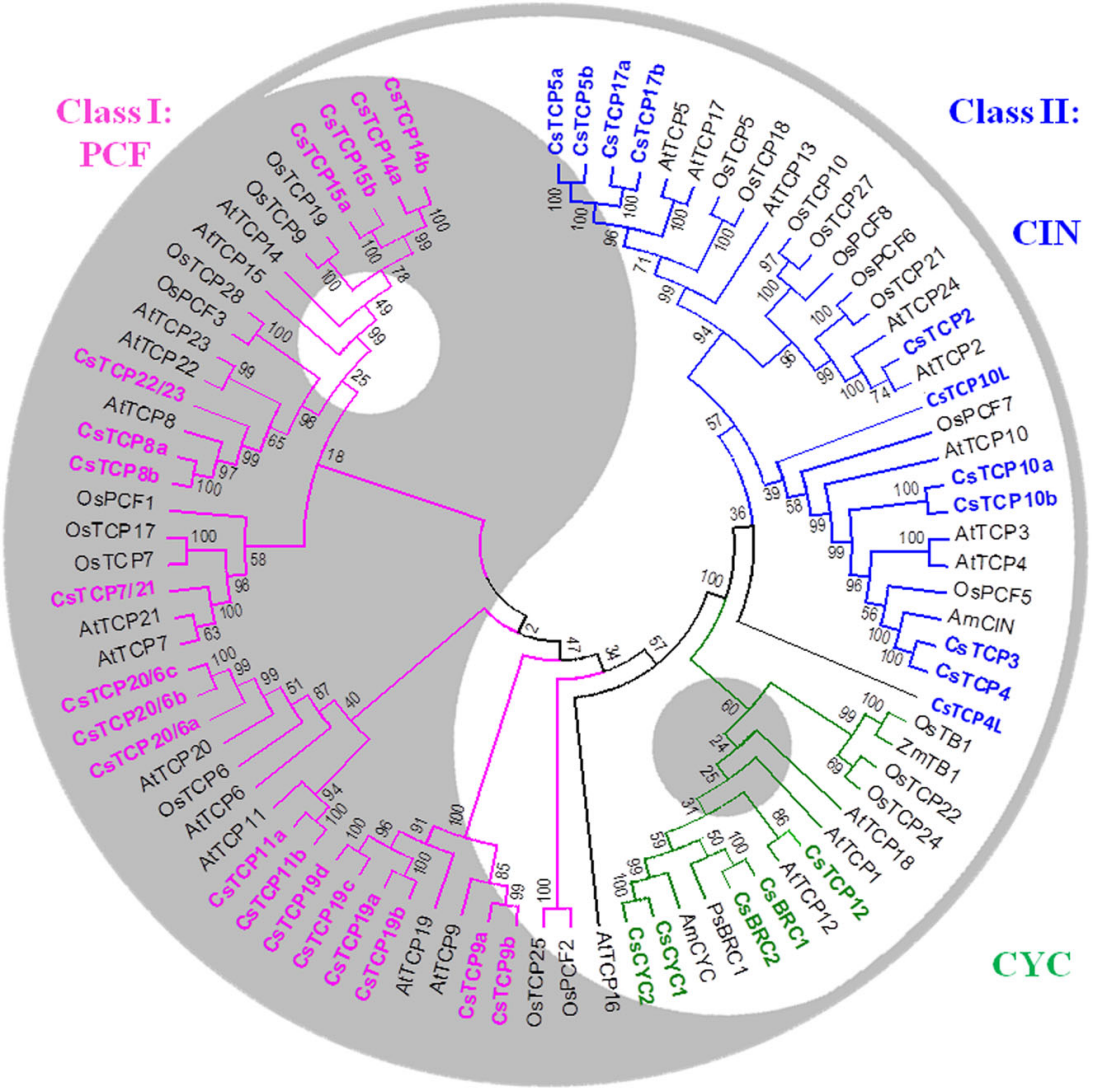
Genome-wide analysis of CsTCP family genes in the tea plant genome. Phylogenetic analysis of CsTCPs from *Camellia sinensis* in comparison with TCPs from Arabidopsis and rice. The amino acid sequences were aligned using ClustalW, and MEGA 6.0 software was used to construct the phylogenetic tree by the NJ method with 1000 bootstrap replicates. The antagonistic but coordinately balanced functions of class-I TCPs and class-II TCPs (including CIN and CYC types) in regulating stem cell division and differentiation during plant tissue and organ growth and development are represented in a Yin (shade) and Yang (light) system

By using Arabidopsis and rice TCP protein sequences as queries for local BLAST searches against the tea plant genome database (http://tpia.teaplant.org/), we identified a total of 35 putative CsTCP proteins that contain conserved TCP domains and whose sequences are most similar to those of Arabidopsis and rice TCP TFs (Fig. 1 and Supplementary Fig. S1)^12^. For our functional study, we annotated tea plant *CsTCP* TFs in accordance with the homology and gene classification terminology used for the Arabidopsis *TCP* family (Supplementary Dataset S1). Essentially, most *C. sinensis CsTCP* TFs have corresponding counterparts in the Arabidopsis genome. However, the tea plant genome apparently contains more *CsTCP* genes than does Arabidopsis. These *CsTCP* genes were predicted to encode TF proteins of 172–544 amino acids with a putative molecular weight (MW) ranging from 19.84 to 60.32 and an isoelectric point (pI) ranging from 5.64 to 10.52 (Supplementary Dataset S1).

Phylogenetic analysis of the CsTCP TF family together with other functionally known Arabidopsis and rice homologs revealed two distinct subfamilies, class-I and class-II TCPs, using NJ tree topology. Class-I CsTCPs are PCF types and include 19 members, and class-II CsTCPs include 16 members referred to as CYC/TB1 and CIN types. The class-II CsTCPs could thus be further divided into subclades CIN (10 members) and CYC/TB1 (6 members) (Fig. 1a and Supplementary Fig. S2a). Multiple sequence alignment of the CsTCP proteins revealed that the basic helix–loop–helix (bHLH) domain-like TCP domain was present in most class-I CsTCPs, excluding CsTCP8a, which does not have a TCP domain but whose sequence is highly similar to that of most TCPs (Supplementary Fig. S1a). A four-amino acid deletion in the TCP domain was found in class-I TCPs compared to the class-II CYC/TB1 and CIN-TCPs. The slightly different but partly overlapping DNA-binding sequences, GGNCCCAC for class I and GTGGNCCC for class-II, are apparent^14^. The R domain, which comprises an ~18-residue arginine-rich motif, is present only in class-II CYC/TB1 proteins at the C-terminus of the TCP domain, with the exception of CsTCP12, which had no R domain (Supplementary Fig. S1b).

Exon/intron analysis showed that the number of exons ranged from one to four in *CsTCP* genes. The majority of *CsTCP* genes contained one exon, and only 5 *CsTCP* genes had two exons, 3 had three exons, and 3 had four exons (Supplementary Fig. S2b). Most *CsTCP* genes clustered together with the Arabidopsis and rice genes in the phylogenetic tree, consistent with the exon/intron structures, indicating the evolutionary conservation of *TCP* gene structure. The conserved motifs were also analyzed, and fifteen motifs in CsTCPs were identified using the MEME program (http://meme-suite.org/) (Supplementary Fig. S2c). All CsTCP family proteins contained motif 1. Motif 15 was present in CsTCPs only of the CIN subclade, including CsTCP5a, CsTCP5b, CsTCP17a, and CsTCP17b. Only PCF-type TFs contained motifs 4, 8, 10, and 14. Motif 6 was conserved only in the CYC subclade, with the exception of CsTCP12. These analysis results suggest that TCP TFs are evolutionarily diverse in *C. sinensis*, which may indicate their largely different roles.

### Expression patterns of *TCP* genes in developing leaves and stems of tea plants

**Fig. 2:**
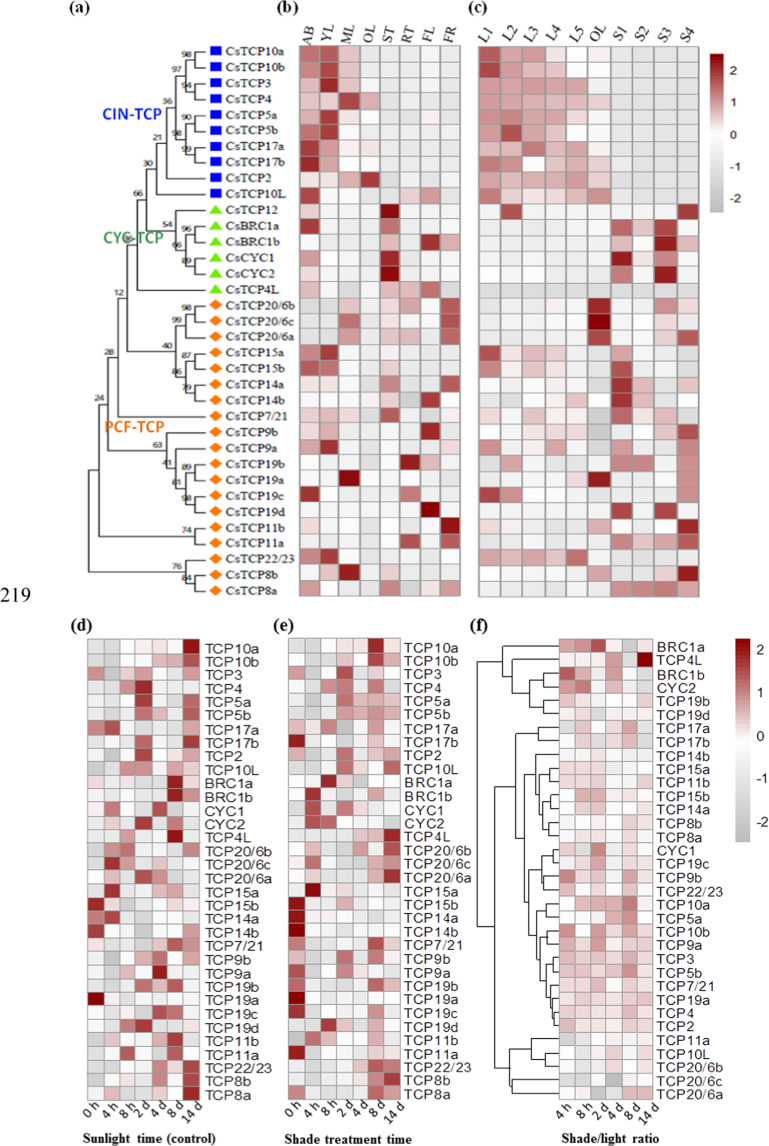
Expression patterns of CsTCP family genes in tea plant tissues at various developmental stages or under light/shade conditions. **a** Phylogenetic classification of CsTCPs from *Camellia sinensi*s. **b** Expression of CsTCPs in different tissues of *Camellia sinensis* plants. Shown are the expression levels of CsTCP genes in eight tissues. AB, apical buds of unopened leaves at the top of actively growing shoots; YL, first and second young leaves below the apical buds; ML, mature leaves geminated in the spring and harvested in the autumn; OL, old leaves at the bottom of tea tree plant; FL, flowers; FR, fruits of tea plants; ST, stem tissues at the 2nd and 3rd internodes; RT, roots. The expression levels were calculated on the basis of log10(FPKM) values. **c** Expression patterns of *CsTCP* genes in developing leaves and stems of tea plants. L1–L5 refer to the 1st, 2nd, 3rd, 4th, and 5th leaves, respectively. **d**, **e** Expression patterns of CsTCPs in tea plants under regular light treatment (**d**) and shade treatment with a black net that allows only 10–20% sunlight to pass (**e**). **f** Comparative expression of tea shoot tips under regular light and shade treatments. The ratio of shade/light is shown in the heatmap analysis

To explore the function of *CsTCP* TFs, we examined the abundance of 35 *CsTCP* transcripts in eight representative tissues of *C. sinensis* cv. Shuchazao (Fig. 2a, b and Supplementary Dataset S2). Interestingly, *CsTCP* genes in every class or subclade displayed distinct and characteristic expression patterns. Most class-II CIN subclade *CsTCP* genes were more highly expressed in the apical bud, young leaf, and mature leaf tissues than in other tissues, consistent with the well-known functions of these genes in regulating leaf development^35^. The expression of these genes either was not detectable or was much lower in the roots, fruits, or stems (Fig. 2b). Most *CYC/TB1* subclade genes were highly expressed in the leaves, stems, and apical buds. For example, *CsTCP12* was highly expressed only in the stems and apical buds and was expressed at relatively low levels in the roots, young leaves, mature leaves, old leaves, flowers, and fruits, strongly suggesting its function in regulating stem branching. The rice *OsTB1*, maize *ZmTB1*, and Arabidopsis *AtBRC1* (*TCP18*) and *AtBRC2* (*TCP12*) homologs are mainly expressed in axillary buds and negatively regulate lateral branching by suppressing axillary bud outgrowth^23,24^. As the expression patterns of these homologous genes (*CsBRC1a* and *CsBRC1b*) in tea plants are similar, CsBRC1s may also have regulatory functions in repressing bud outgrowth (Fig. 2b) The tea plant genes *CsBRC1a*, *CsBRC1b*, *CsCYC1*, and *CsCYC2* are highly expressed in the stems (1st, 2nd, and 3rd stems); *CsBRC1a* and *b* also show expression at relatively high levels in fruits and flowers (Fig. 2b).

Class-II TCPs are generally thought to act as repressors of cell division and inducers of cell differentiation^6,7^. The dynamic expression of *CIN-*type *TCP* genes affects leaf shape by regulating the timing of leaf maturation^36,37^. Mutation of the *CIN-TCP* gene LANCEOLATE (LA) in tomato (*Solanum lycopersicum*) or Arabidopsis alters leaf growth and maturation, leading to curled, crinkled, or rolled leaves due to overgrowth of and cell proliferation in certain leaf areas^19,20,37,38^. Like their Arabidopsis counterparts AtTCP3, -4, and -10, CIN-type *CsTCP*s (specifically *CsTCP2*, *-3*, and -*4*; *CsTCP5a* and *CsTCP5b*; *CsTCP17a* and *CsTCP17b*; and *CsTCP10a* and *CsTCP10b*) are expressed in apical buds and young leaves. These TCPs likely act as negative regulators of leaf development, playing a pivotal role in the control of morphogenesis by negatively regulating the expression of boundary-specific genes^18^.

Class-I PCF *TCP* genes displayed more extensive tissue-specific expression patterns. The expression patterns of *AtTCP7*, *AtTCP8*, *AtTCP22*, and *AtTCP23* are similar in young Arabidopsis leaves, and these genes are functionally redundant with respect to leaf development regulation^38,39^. In tea plants, both *CIN-*type and some *PCF-*type *TCP* genes are highly expressed in apical buds and developing leaves. AtTCP15 regulates the expression of boundary-specific genes, and the function of this protein partially overlaps with the functions modulated by class-II CIN-like TCP proteins with opposite effects^40^. AtTCP14 and AtTCP15 redundantly regulate internode development by promoting cell proliferation^25,26^. *CsTCP15a*, *CsTCP15b*, *CsTCP9b*, *CsTCP19b*, *CsTCP19c*, and *CsTCP7* were highly expressed in both young leaves and mature leaves. In addition, *CsTCP7/21* and *CsTCP8b* were highly expressed in the stem, young leaf, and mature leaf tissues, whereas *CsTCP14a* and *CsTCP22/23* were highly expressed in the apical bud and young leaf tissues. We also observed that *CsTCP19a*, *CsTCP20a*, *CsTCP20b*, and *CsTCP20c* were highly expressed in old leaves and that the expression of *CsTCP19a* was upregulated in response to MeJA treatment (Supplementary Dataset S3 and S6).

### Expression patterns of *CsTCP*s in response to hormones and shade treatment

A large number of *cis*-acting elements involved in plant growth and development and hormone responses were identified in the promoters of *CsTCP* genes (Supplementary Fig. S4 and Supplementary Dataset S4). The *cis*-elements in the 1.50 kb promoter region of each *CsTCP* gene contained various types of *cis*-acting elements (Supplementary Dataset S4). The regions included elements responsive to light (37%), hormones (27.8%), environmental stress (25.50%), and plant growth (9.70%) (Supplementary Fig. S4a). Along with these reported regions, there were some TF-binding sites that were also found to be responsive to hormones such as abscisic acid (ABA), GA, auxin, JA, salicylic acid, and ethylene (Supplementary Fig. S4b) or to stresses such as pathogen infection, heat, low temperature, and drought (Supplementary Fig. S4c, d). The expression of all development-related T*CP TF* genes, such as *CsTCP2*, *CsTCP10a*, *CsTCP3*, *CsTCP20/6c*, *CsTCP14a*, *CsTCP22/23*, and *CsTCP8a*, was repressed by drought and NaCl stresses (Supplementary Fig. S5a). The expression levels of most *CsTCP*s were unchanged or slightly repressed, and the expression of only *CsTCP10a*, *CsTCP11b*, and *CsTCP5a* was upregulated by MeJA treatment (Supplementary Fig. S5a–c). The patterns of *cis*-acting elements differed among *CsTCP* members (Supplementary Dataset S4). The promoter of *CsTCP4* contains the maximum number (18) of hormone-responsive elements recorded, followed by the promoters of *CsTCP11a* (15) and *CsTCP14b* (14) (Supplementary Dataset S4). The *CsTCP4* and *CsTCP10b* promoters contain the most (6) ABA-responsive elements, while the promoters of *CsTCP9a* and *CsTCP14b* contain at most (6) ERE motifs (Supplementary Dataset S4). *CsTCP4* also contains (5) TCA elements that are related to the salicylic acid response. Most of the *CsTCP*s, namely, *CsTCP9*, *-10*, and -*11*, contained an equal number of environmental stress-responsive elements, but *CsTCP14b* contained the minimum number of environmental stress-responsive elements (only 2) (Supplementary Dataset S4). *CsTCP19b* contained the most (11) MYB-related elements (Supplementary Dataset S4).

**Fig. 3:**
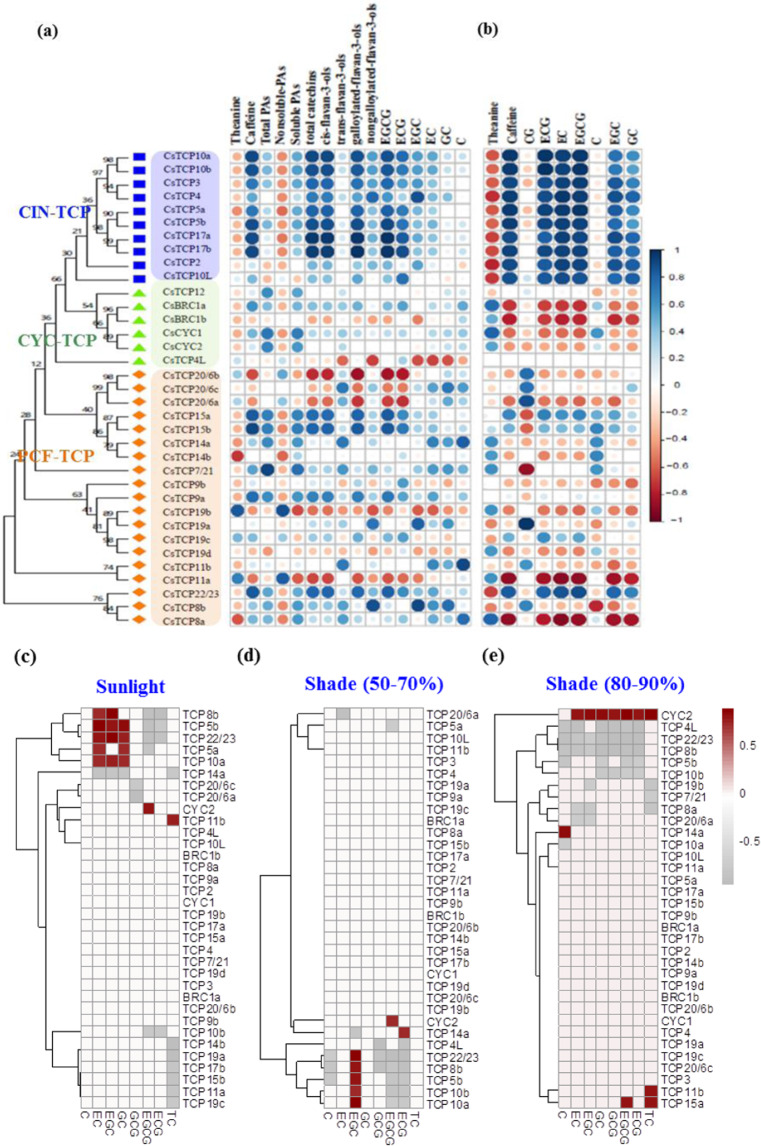
Correlation analysis of *CsTCP* genes with the accumulation of major tea plant secondary metabolites. **a**, **b** Integrative analysis of CsTCP expression and secondary metabolite accumulation patterns in eight representative tissues of tea plants (**a**) or in six representative leaf tissues and four representative stem tissues at various developmental stages (**b**). Correlation analysis conducted with 16 metabolites and 35 transcripts of *CsTCP*s. *R* > 0.5: Positive correlations; *R* < −0.5: negative correlation. **c**–**e** Correlation analysis of CsTCP expression with secondary metabolite accumulation in shoot tips under regular sunlight treatment (**c**), shade treatment (50–70% of transmitted sunlight blocked) (**d**), and shade treatment (80–90% of transmitted sunlight blocked) (**e**). The shoot tip (including apical buds and the 1st leaf) tissues of the Shuchazao variety were treated with shading as previously described (Liu et al., 2018). Shoot tips of tea plants under regular sunlight were set as controls, and four representative stem tissues at various developmental stages were used

Light is a key factor affecting plant growth, development, and metabolism. PHYTOCHROME INTERACTING FACTORs (PIFs) are key factors involved in the light signaling network^41^. AtTCP5, AtTCP13, and AtTCP17 were reported to be involved in plant responses to light, temperature, and shade in both PIF-dependent and PIF-independent pathways^42^. Our previous study showed that shade treatment drastically affects tea plant shoot tip growth, morphology, and secondary metabolism^43^. Under shade treatment, the expression levels of the CIN-type genes *CsTCP5a*, *CsTCP5b*, and *CsTCP10a* were upregulated at 8 d, and the expression of *CsTCP10b* was upregulated at 4 h and 2 d. The expression of the CYC-type *CsBRC1a* gene was upregulated significantly at 2 d, and the expression of the *CsBRC1b* and *CsCYC2* genes was upregulated significantly at 4 h and 8 h but then repressed at 14 d. Similarly, the expression of *CsTCP9a* and *CsTCP9b* was upregulated at 2 d and 4 h, respectively (Fig. 2f and Supplementary Dataset S5). AtTCP17 interacts with the blue light receptor CRYPTOCHROME 1 (CRY1) at low temperatures, leading to reduced TCP1742 activity^42^. Under shade treatment, the expression levels of *CsTCP5a*, *CsTCP5b*, and *CsTCP17b*, together with their putative target *CsPIF4*, and another *CsTCP TF*, *CsTCP10a*, were significantly upregulated 8 d after shading compared to normal light. Similar to AtTCP14/15 inhibition of anthocyanin accumulation during exposure of plants to high-light intensity^29^, the *CsTCP14a*, *CsTCP15a*, and *CsTCP15b* gene expression patterns were consistent with the total catechin content (Fig. 3a).

### Relationship between *CsTCP* expression and metabolite accumulation

Transcriptome and metabolome profiling of eight representative tissues [buds, leaves, stems, roots, flowers, fruits, leaves at six different developmental stages, and four internodes (stems) from the shoot tip to lower levels of tea plants] were used to determine the possible correlations between *CsTCP* TFs and tea plant secondary metabolite biosynthesis. Through an integration of the metabolite profiling and transcriptome data, a gene-to-metabolite correlation analysis of 16 flavonoid metabolites, including total catechins, caffeine, and theanine, with the expression patterns of all *CsTCP* genes in these specific tissues was conducted (Fig. 3a). The expression patterns of *CsTCP2*, -*3*, -*4*, -*5*, -*10*, and -*17* were positively correlated with gallocatechin (GC), epigallocatechin (EGC), epigallocatechin gallate (EGCG), epicatechin (EC), epicatechin gallate (ECG), and caffeine contents but negatively correlated with theanine contents (Fig. 3a). The PCF-type *CsTCP22/23* was also positively correlated with the GC, EGC, EGCG, EC, ECG, and caffeine contents in developing leaves and stems (Fig. 3a). The expression patterns of CYC-type *CsTCP* genes were not significantly correlated with the metabolite levels in the eight tissues (Fig. 3a). However, in the developing leaves and stems at various stages, CYC-type *CsTCP* genes (except for *CsTCP12*) and PCF-type *CsTCP*s, such as *CsTCP11a* and *CsTCP8a*, showed a clearly negative correlation with these metabolites (Fig. 3b).

PCF-type *CsTCP20/6b*, *CsTCP20/6a*, and *CsTCP11a* expression was negatively correlated with galloylated catechins (ECG, EGCG, and *cis*-flavan-3-ols), total catechins, and caffeine. *CsTCP15a* and *CsTCP15b* expression was positively correlated with the contents of galloylated catechins (ECG, EGCG, CG), total catechins, and caffeine (Fig. 3a). Their opposite correlations with catechins were similar to those concerning theanine contents in these tissues. Moreover, *CsTCP14b* expression showed a clearly negative correlation with theanine, but *CsTCP19b* expression had a positive correlation with theanine (Fig. 3a). Specifically, *CsTCP7/21* expression was negatively correlated with CG level; *CsTCP20/6b*, *CsTCP20/6a*, *CsTCP20/6c*, and *CsTCP19b* expression was positively correlated with CG content; *CsTCP8b* expression was negatively correlated with C contents; and *CsTCP4* expression seemed to be closely correlated with C contents (Fig. 3b). Moreover, the CIN-type and CYC-type class-II *TCP* genes showed opposite correlations with the accumulation of catechins in the leaf and stem tissues at various developmental stages.

Our previous study showed that shade treatment of tea plant shoot tips enhanced chlorophyll accumulation but significantly decreased the contents of major catechins, including C, EC, GC, and EGC, under shade treatment compared with light treatment^43^. The total catechin contents displayed a significant decrease from 4 h to 14 d in the S80–90% treatments, and the ECG content decreased until 14 d of treatment, whereas the GCG and EGCG contents showed only a minor decrease in the shade treatment^43^. Under regular light conditions, the expression patterns of *CsCYC2* were correlated with EGCG content variations over two weeks. Similarly, *CsTCP11b* expression was correlated with total catechins, whereas the expression of *CsTCP8b*, *CsTCP5a*, *CsTCP5b*, *CsTCP10a*, and *CsTCP22/23* was correlated with content variations of at least two of ECs, EGCs, and GCs (Fig. 3c). However, under the shade treatment that blocked 50–30% of sunlight through the net, the EGC content variations in tea plant shoot tips (apical buds and the first fully open leaf) were significantly correlated with the expression of *CsTCP8b*, *CsTCP5b*, *CsTCP10a*, *CsTCP10b*, and *CsTCP22/23*. Moreover, *CsCYC2* was also correlated with EGCG contents, and *CsTCP14a* was correlated with ECG contents (Fig. 3d). Under the shade treatment that allowed only 10–20% of sunlight to pass through the net, *CsCYC2* expression was also correlated with most types of catechins, and the total catechin content, except for C, and *CsTCP14a* expression was correlated with the C content. In addition, *CsTCP11b* and *CsTCP15a* expression was correlated with total catechins, and *CsTCP15a* expression was also significantly correlated with EGCG content (Fig. 3e). *CsTCP7/21* expression was weakly induced under 80–90% shading (Fig. 2f), which may enhance chlorophyll accumulation. In MeJA-treated shoot tips, the expression levels of *CsTCP5a*, *CsTCP5b*, *CsTCP10a*, *CsTCP11a*, *CsTCP17a*, *CsCYC2*, *CsTCP3*, *CsTCP19a*, and *CsTCP14b* were upregulated compared with those of the controls (Supplementary Dataset S6).

### CsTCPs are involved in the regulation of catechin biosynthesis

**Fig. 4:**
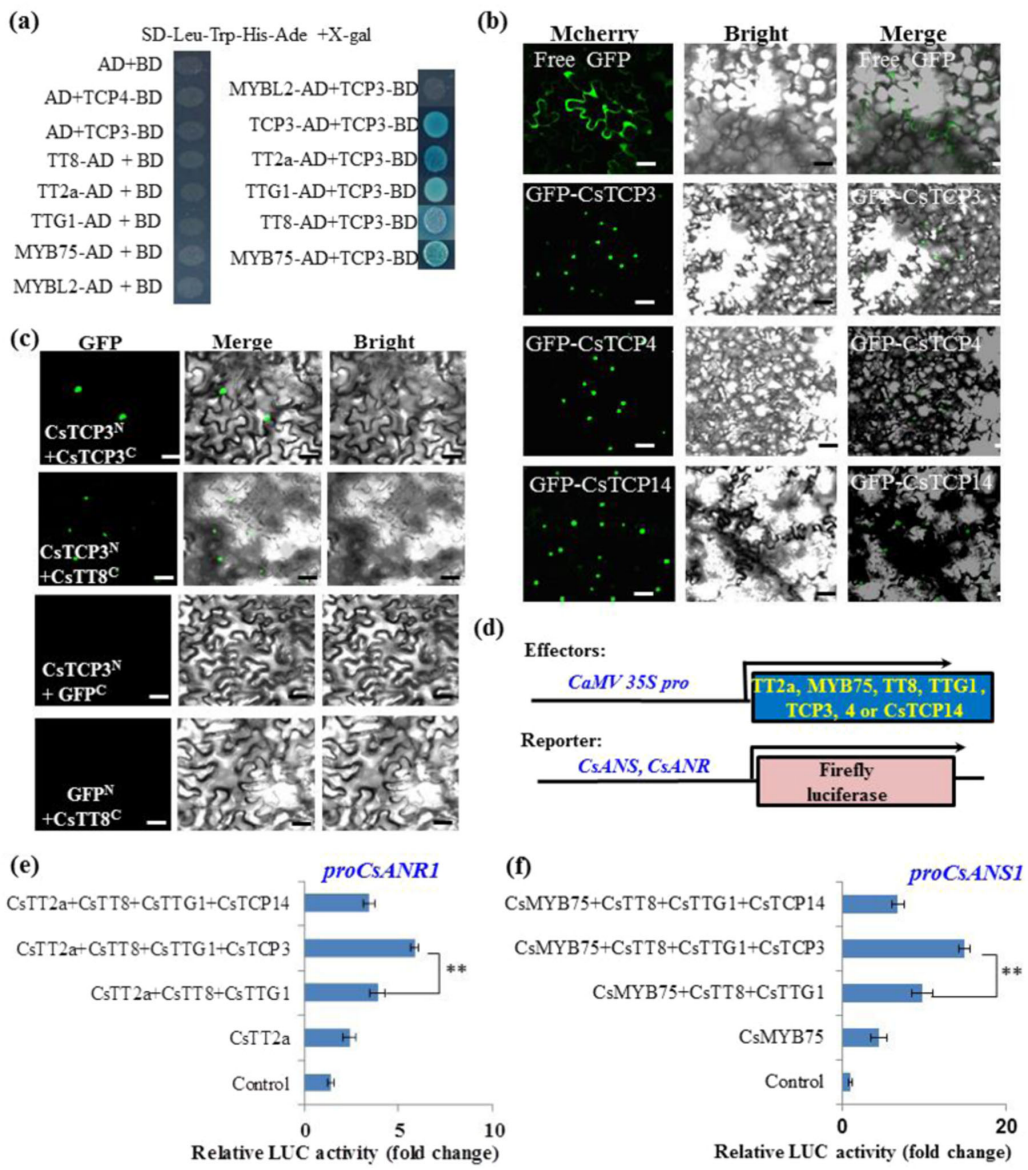
Involvement of CsTCPs in the regulation of flavonoid biosynthesis through a ternary regulatory complex. **a** Interactions between CsTCP3 and three components of the MBW complex, as revealed by a Y2H assay. **b** Nuclear localization of GFP-CsTCP3, GFP-CsTCP4, and GFP-CsTCP14 fusion proteins in tobacco leaf epidermal cells. Bar = 60 µm. **c** BiFC verification of the formation of homodimers or heterodimers between CsTCP3 and CsTT8a. **d** Construction of effectors and reporter vectors for a transactivation assay with the promoters of *CsANS1* and *CsANR1*. **e**, **f** Transactivation assay of CsTCP3 and CsTCP4 together with the MBW complex on the promoter of *CsANR1* (**e**) and *CsANS1* (**f**). The data were from three independent experiments and expressed as the means ± SDs (*n* = 3). The differences from the controls were analyzed by two-tailed comparisons. *P < 0.05; **P < 0.01 according to Student’s *t*-test

To verify the functions of CsTCPs in the regulation of tea plant secondary metabolism, we further studied the regulatory functions of the CIN-type TCP TFs CsTCP3 and CsTCP4, as well as PCF-type CsTCP14, in flavonoid biosynthesis. Yeast two-hybrid assays indicated that CsTCP3 and -4 interacted with several flavonoid regulators, including the anthocyanin-specific MYB regulator CsMYB75, the proanthocyanidin (PA)-specific regulators CsTT2a, CsTTG1, and CsTT8, and a negative regulator of anthocyanin CsMYBL2. Moreover, the bHLH TFs CsTCP3 and CsTCP4 interact with themselves to form homo- and heterodimers, which is consistent with their interactions with CsTT8 to form heterodimers (Fig. 4a). Furthermore, bimolecular fluorescence complementation (BiFC) assays in tobacco cells also confirmed that CsTCP3 N-GFP interacted with CsTT8a-C-GFP to form a heterodimer in planta (Fig. 4c). These data suggest that these MYBs, bHLHs, and WD40 proteins could interact, likely forming an MBW complex, similar to previously reported phenomena in other plant species^44^. To test this hypothesis, we examined whether these interacting TFs could form an active regulatory complex by using a transactivation assay with a dual-luciferase reporter system^44^. Transactivation assays using tea plant anthocyanidin synthase (*CsANS1*) and anthocyanidin reductase (*CsANR1*) promoters showed that adding CsTCP3 to the MBW complex activation system further enhanced *CsANS* and *CsANR* promoter activities. However, the addition of CsTCP14 to the promoter activation system inhibited the MBW-induced activation of the *CsANS1* and *CsANR1* promoters (Fig. 4d–f). Since CsTT2a or CsMYB75 could form the MYB-bHLH-WD40 (MBW) regulatory complex to regulate PA and anthocyanin biosynthesis in tea plants, our data are in line with those of a previous report showing that AtTCP3 regulates flavonoid biosynthesis^34^.

### *CsTCP*s and *microRNA*s regulate tea plant leaf development

**Fig. 5:**
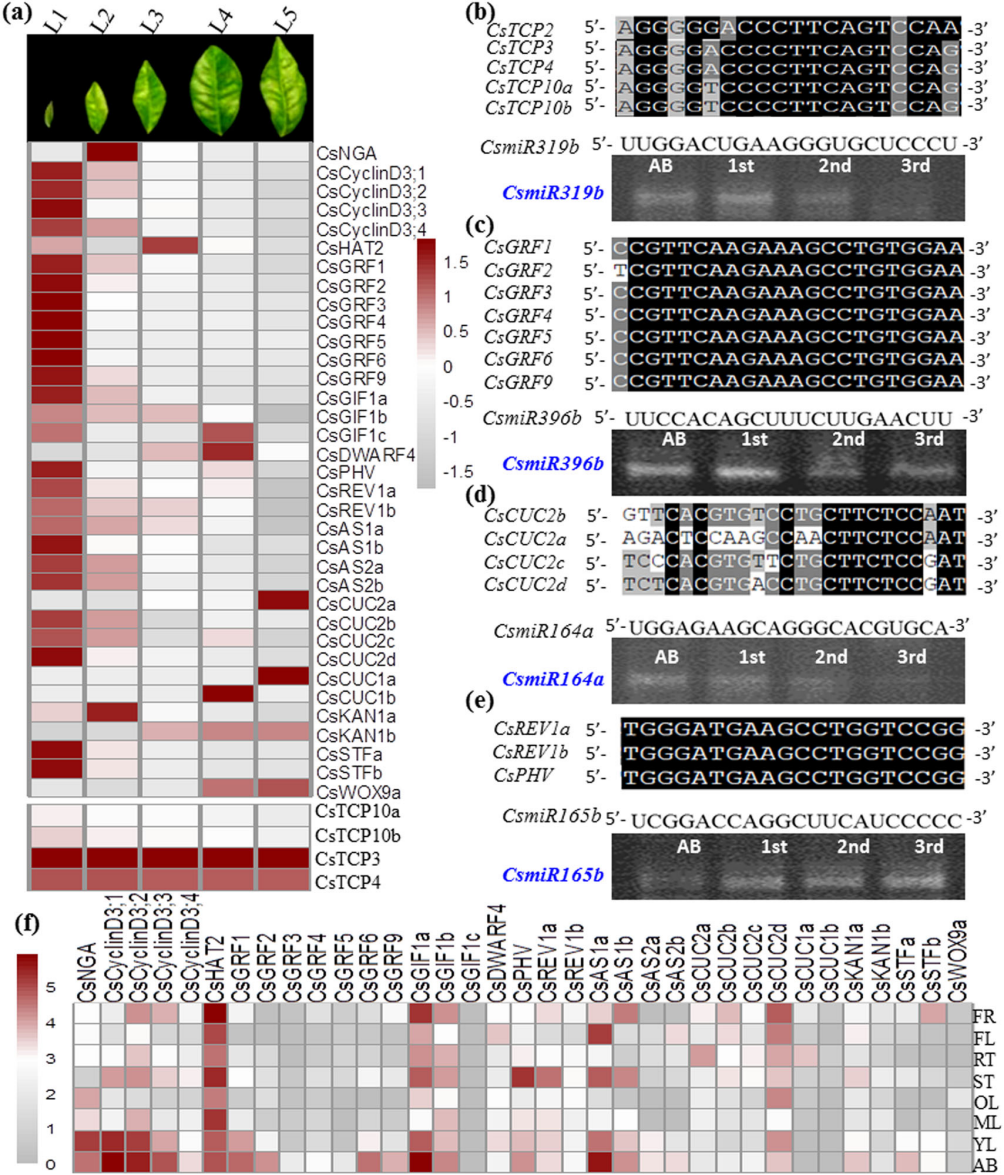
Expression patterns of miR319-CsTCP and other miRNA modules in the developing leaves of tea plants. **a** Expression of development-related genes at different stages of tea plant leaf or stem growth and development in comparison with the expression of *CsTCP3*, *CsTCP4*, *CsTCP10a*, and *CsTCP10b*. **b** Top panel: alignment of *CsmiR319b* with its target genes *CsTCP2* (1048–1067 bp), *CsTCP3* (922–941 bp), *CsTCP4* (1345–1364 bp), *CsTCP10a* (772–79 bp), and *CsTCP10b* (1396–1415 bp), which are complementary to *CsmiR319b* (1–21 pb). Bottom panel: RT-PCR-based assessment of CsmiR165b transcripts in apical buds (AB) and in the 1st, 2nd, and 3rd leaves from the shoot tips. A representative image is shown. **c** Top panel: alignment of *CsmiR396b* with its target genes *CsGRF1* (870–891 bp), *CsGRF2* (755–776 bp), *CsGRF3* (342–363 bp), *CsGRF4* (348–369 bp), *CsGRF5* (339–360 bp), *CsGRF6* (360–381 bp), and *CsGRF9* (420–441 bp), which are complementary to *CsmiR396b* (1–21 bp). Bottom panel: RT-PCR-based assessment of *CsmiR396b* transcripts in apical buds (AB) and in the 1st, 2nd, and 3rd leaves from the shoot tips. A representative image is shown. **d** Top panel: alignment of *CsmiR164b* with its target genes *CsCUC2a* (454–475 bp), *CsCUC2b* (637–660 bp), *CsCUC2c* (619–642 bp), and *CsCUC2d* (652–675 bp), which are complementary to *CsmiR164b* (1–21 bp). Bottom panel: RT-PCR-based assessment of *CsmiR164b* transcripts in apical buds (AB) and in the 1st, 2nd, and 3rd leaves from the shoot tips. A representative image is shown. **e** Top panel: alignment of *CsmiR165b* with its target genes *CsREV1a* (564–583 bp), *CsREV1b* (939–958 bp), and *CsPHV* (567–586 bp), which are complementary to *CsmiR165b* (1–19 bp). Bottom panel: RT-PCR-based assessment of *CsmiR165b* transcripts in apical buds (AB) and in the 1st, 2nd, and 3rd leaves from the shoot tips. A representative image is shown. **f** Expression patterns of development-related genes in different tissues of tea plants

We further identified several sets of genes that have been demonstrated to be critical players in regulating leaf and shoot development in Arabidopsis and other plant species. These included the following: genes that encode GRFs and GIFs that positively promote leaf cell proliferation^6,7,45^; CUCs that regulate embryonic shoot meristem and leaf boundary formation^18,46^; the HD-ZIPIII proteins PHAVOLUTA (PHV) and REVOLUTA (REV), which are required to establish leaf adaxial identity and patterning; and other related genes, such as those that encode cell division-related proteins including Cyclin Ds, ASYMMETRIC LEAVES 1/2 (AS1/2), and HOMEODOMAIN LEUCINE ZIPPER 2 (HAT2)^31,32,36^ (Fig. 5a, Supplementary Datasets S8 and S9, and Figs. S7–10).

Many miRNAs have been identified from tea plant microRNA transcriptome studies^47^, and they are essentially conserved in other plant species^22,30,33^ (Supplementary Dataset S7). Five CIN-type *CsTCP* genes (*CsTCP2*, -*3*, *4*, -*10a*, and -*10b*) contain a putative *miR319* target site (Fig. 5b), suggesting that the *CsmiR319*/*CsTCP* module may be conserved in terms of its ability to regulate leaf development in *C. sinensis*. *CsmiR319* transcript levels decreased as the leaf aged during development, since expression in the 3rd leaf was much lower than that in the 2nd or 1st leaf (Fig. 5b). From the miRNA database, we also identified a *CsmiR396b* homolog whose sequence was complementary to the target sequence sites of the CsGRF and CsGIF genes in tea plant (Fig. 5c, Supplementary Figs. S7–10 and Supplementary Dataset S7). The *CsmiR396b* transcript level also slightly decreased with leaf age (Fig. 5c). In addition, these CIN-type *TCP*s also induced the expression of *AS1* and *AS2*, which are involved in adaxial polarity patterning, and of *miR164*, which targets *CUC* genes involved in the regulation of leaf boundary region development (Fig. 5d). *CsmiR164a* expression was the lowest recorded in the 3rd leaf, indicating a decrease with leaf age (Fig. 5d). *AS1/2* regulates the expression of *miR165*, which cleaves its target HDIII genes, *CsPHV* and *CsREV1a* (Fig. 5e). The levels of *CsmiR165b* transcripts, in contrast, increased with leaf age, as the highest levels were detected in the 3rd leaf (Fig. 5e).

**Fig. 6:**
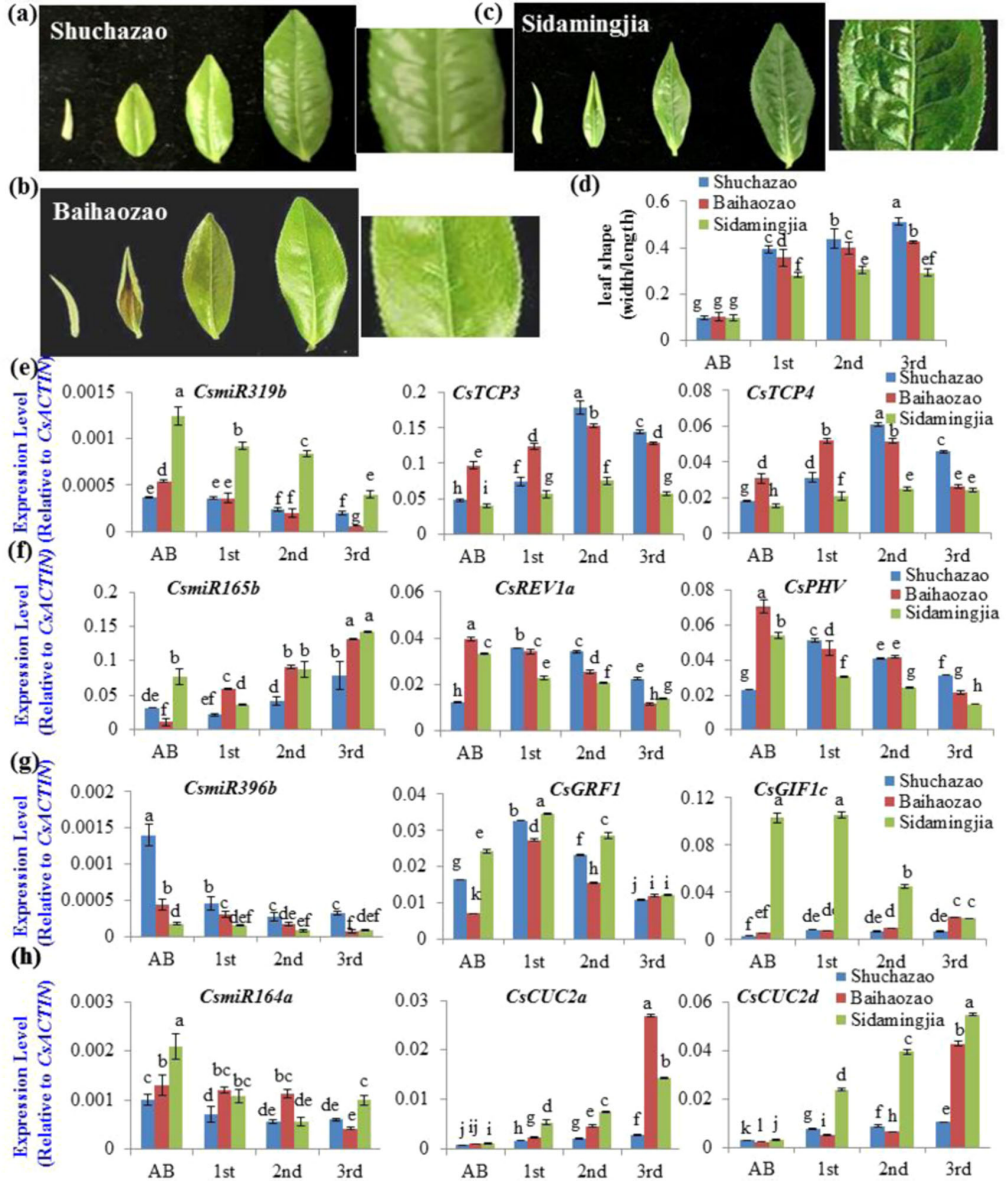
Involvement of *miR319/CsTCP* and other miRNA/target modules in leaf development of different tea plant varieties. **a**–**c** Leaf shape and patterns of the apical bud (AB) and the 1st, 2nd, and 3rd leaves of three tea plant varieties Shuchazao (**a**), Baihaozao (**b**), and Sidamingjia (**c**). **d** Ratios of leaf width to length of leaves from the apical bud (AB) and 1st, 2nd, and 3rd leaves of three tea plant varieties. **e** Expression patterns of *CsmiR319b* with its target genes *CsTCP3* and *CsTCP4* in the apical bud (AB) and 1st, 2nd, and 3rd leaves. **f** Expression patterns of *CsmiR165b* with its target genes *CsREV1a* and *CsPHV* in the apical bud (AB) and 1st, 2nd, and 3rd leaves. **g** Expression patterns of *CsmiR396b* with its target genes *CsGRF1* and *CsGIF1c* in the apical bud (AB) and 1st, 2nd, and 3rd leaves. **h** Expression patterns of *CsmiR164b* with its target genes *CsCUC2a* and *CsCUC2d* in the apical bud (AB) and 1st, 2nd, and 3rd leaves. The relative expression levels of the miRNA genes were measured via qRT-PCR and normalized to the average expression level of *CsACTIN*. The data are expressed as the means ± SDs of three independent experiments, each with multiple biological replicates. Differences between tea plant varieties were analyzed with two-factor ANOVA using the LSD_0.05_ method

We examined the expression patterns of *miRNA*s and their target genes in three varieties with distinct leaf shapes during leaf development (Fig. 6d). Shuchazao plants have round 1st leaves (Fig. 6a); Baihaozao plants have slightly long and curled 1st leaves (Fig. 6b); and Sidamingjia plants have long, narrow, curled 1st leaves (Fig. 6c). In addition, the 2nd and 3rd leaves of the Baihaozao variety were flat, with no clear crinkled leaf surface between veins (Fig. 6b). However, Sidamingjia has curled or crinkled leaves (Fig. 6c), and Shuchazao also has moderately curled or crinkled leaves (Fig. 6a). They all have jagged leaf edges.

Interestingly, a similarly decreasing pattern was observed for *miR319* transcripts from the apical bud to the 3rd leaf in all three tea plant varieties (Fig. 6e). However, the Shuchazao leaves had a much lower *miR319* expression level, approximately one-third of that in Sidamingjia leaves, while Baihaozao 1st leaves presented approximately one-half of that presented by the Sidamingjia leaves. Correspondingly, the transcripts of *CsTCP3* and *CsTCP4* gradually increased with leaf age in all three varieties (Fig. 6e). The transcript levels of *CsTCP3* and *CsTCP4* in Sidamingjia leaves were 2- to 4-fold lower than those in Shuchazao and Baihaozao leaves. These results suggest that *CsmiR319b* is likely involved in the cleavage of *CsTCP3* and *CsTCP4* transcripts.

The *miR165/166* targets *CsPHV* and *CsREV1a*, *miR164/CUC*s, and *miR396/GRF-GIF* modules were examined (Fig. 6f–h). The expression of *CsmiR164a* displayed similar patterns: it steadily decreased in apical buds and the 1st, 2nd, and 3rd leaves of Shuchazao and Baihaozao but continuously increased in Sidamingjia (Fig. 6h). In round-leaf Shuchazao plants, the overall expression levels of *miR164a* in the apical buds and developing leaves were approximately half of those of the long, narrow-leaf varieties Sidamingjia and Baihaozao. Correspondingly, the transcripts of *miR164a* target genes such as *CsCUC2a* and *CsCUC2d*, two major *CUC* genes highly expressed in 3rd leaves, were also at much higher levels in Sidamingjia than in Shuchazao and Baihaozao.

CUC1-3 NAC TFs form a critical boundary domain that delimits leaflets^48^. These TFs promote leaf marginal outgrowth, local leaflet separation, and distal leaflet formation^48–50^. By contrast, reduced *CUC1-3* gene expression leads to fewer and fused leaflets. Relatively low expression levels of *CsCUC2a* and *CsCUC2d* genes in Shuchazao leaves partly explained the more extended outgrowth of leaflets, whereas Baihaozao and Sidamingjia had relatively high expression levels of *CsCUC2a* and *CsCUC2d*, coinciding with their narrow and long 1st leaves (Fig. 6h).

The *miR165b* transcripts in all three varieties showed similarly increasing patterns from ABs to the 1st, 2nd, and 3rd leaves. However, *miR165b* transcript levels were generally higher in Sidamingjia and Baihaozao buds and young leaves than in those of Shuchazao (Fig. 6f). Their target genes, *CsREV1a* and *CsPHV*, showed opposite changes in expression: their expression decreased slowly throughout leaf development. These target genes are required to establish adaxial identity in plant lateral organ primordia. Both *CsREV1a* and *CsPHV* transcript levels decrease throughout leaf development more rapidly in the long, narrow-leaf varieties Sidamingjia and Baihaozao than in the round-leaf variety Shuchazao, suggesting that *miR165b* modulates leaf shape regulation in these three tea varieties.

The *miR396b* transcripts rapidly decreased from the highest level in the apical buds to the lowest levels in the 2nd and 3rd leaves. The level of *miR396b* was significantly higher in the Shuchazao buds and leaves—approximately 3- and 9-fold higher than in those of Baihaozao and Sidamingjia, respectively (Fig. 6g). This was consistent with rapidly increasing expression levels of cell growth- and proliferation-promoting genes, such as *CsGRF1* and *CsGIF1c*. *CsGRF1* was also expressed at much higher levels in Shuchazao than in Baihaozao.

**Fig. 7:**
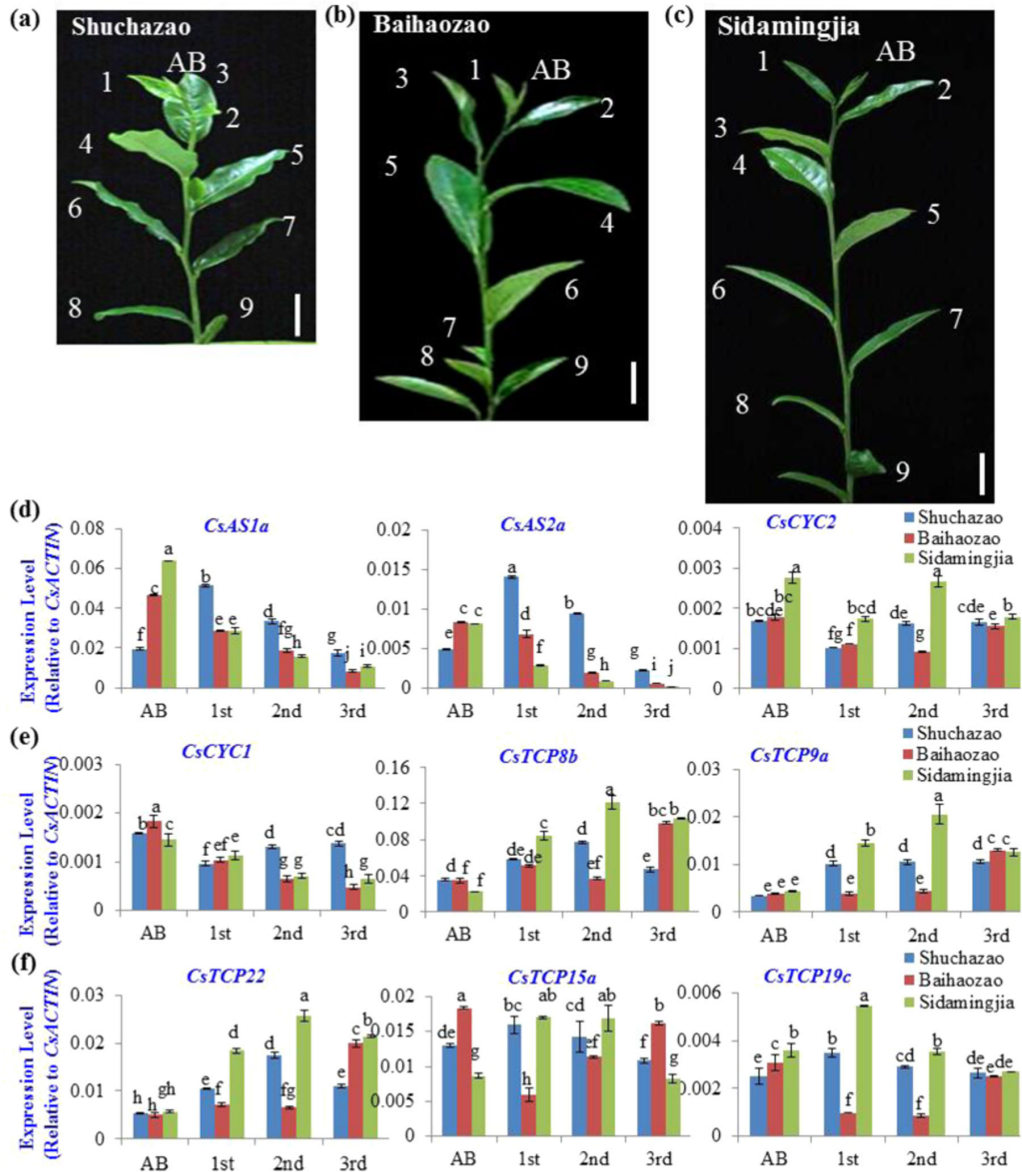
Involvement of *miR319-CsTCP* and other genes in leaf development of tea plant variety. **a**–**c** Shoot tip of tea plant varieties Shuchazao (**a**), Baihaozao (**b**), and Sidamingjia (**c**). From the top are the apical buds (ABs) and the 1st, 2nd, 3rd, 4th, 5th, 6th, 7th, 8th, and 9th leaves. The short, middle, and long internodes are shown. Bar = 1 cm. **d** Expression patterns of *CsAS1*, *CsAS2*, and *CsCYC2* in the apical bud (AB) and 1st, 2nd, and 3rd leaves of three tea plant varieties. **e** Expression patterns of *CsCYC1*, *CsTCP8b*, and *CsTCP9a* in the apical bud (AB) and 1st, 2nd, and 3rd leaves of three tea plant varieties. **f** Expression patterns of *CsCTCP22*, *CsTCP15a*, and *CsTCP19c* in the apical bud (AB) and 1st, 2nd, and 3rd leaves of three tea plant varieties. The relative expression level of the *CsmiR319b* gene was measured via qRT-PCR and normalized to the average expression level of *CsACTIN*. The data are expressed as the means ± SDs of three independent experiments, each with multiple biological replicates. Two-factor ANOVA was performed on the data, and the differences were analyzed using the LSD_0.05_ method

The transcript levels of two developmental repressors, *CsAS1a* and *CsAS1b*, continuously decreased in the 1st, 2nd, and 3rd leaves, with relatively higher levels detected in Shuchazao than in Baihaozao and Sidamingjia (Fig. 7d). The transcripts of two CYC-type *TCP*s, *CsCYC1* and -*2*, also decreased throughout leaf development. While Sidamingjia had higher *CsCYC2* expression levels, Shuchazao had higher *CsCYC1* expression levels in the 2nd and 3rd leaves (Fig. 7d, e). Five class-I *TCP* genes were highly expressed in young leaves and may play roles in regulating leaf development. The transcript levels of most of these *CsTCP*s, e.g., *CsTCP8b*, *CsTCP9a*, *CsTCP22*, *CsTCP15a*, and *CstCP19c*, increase during leaf growth and development to different extents. We found that the expression of most of these genes in the 1st and 2nd leaves was higher in Shuchazao and Sidamingjia than in Baihaozao; however, in the 3rd leaf, most PCF-type genes (*CsTCP8b*, *CsTCP9a*, and *CsTCP22*) were expressed at higher levels in Baihaozao and Sidamingjia. *CsTCP15a* was expressed at a higher level in the 3rd leaf of Baihaozao than in those of the other two varieties (Fig. 7e, f).

### The *CsmicroR319b/CsTCP3-4* module affects catechin biosynthesis in tea plant leaves

**Fig. 8:**
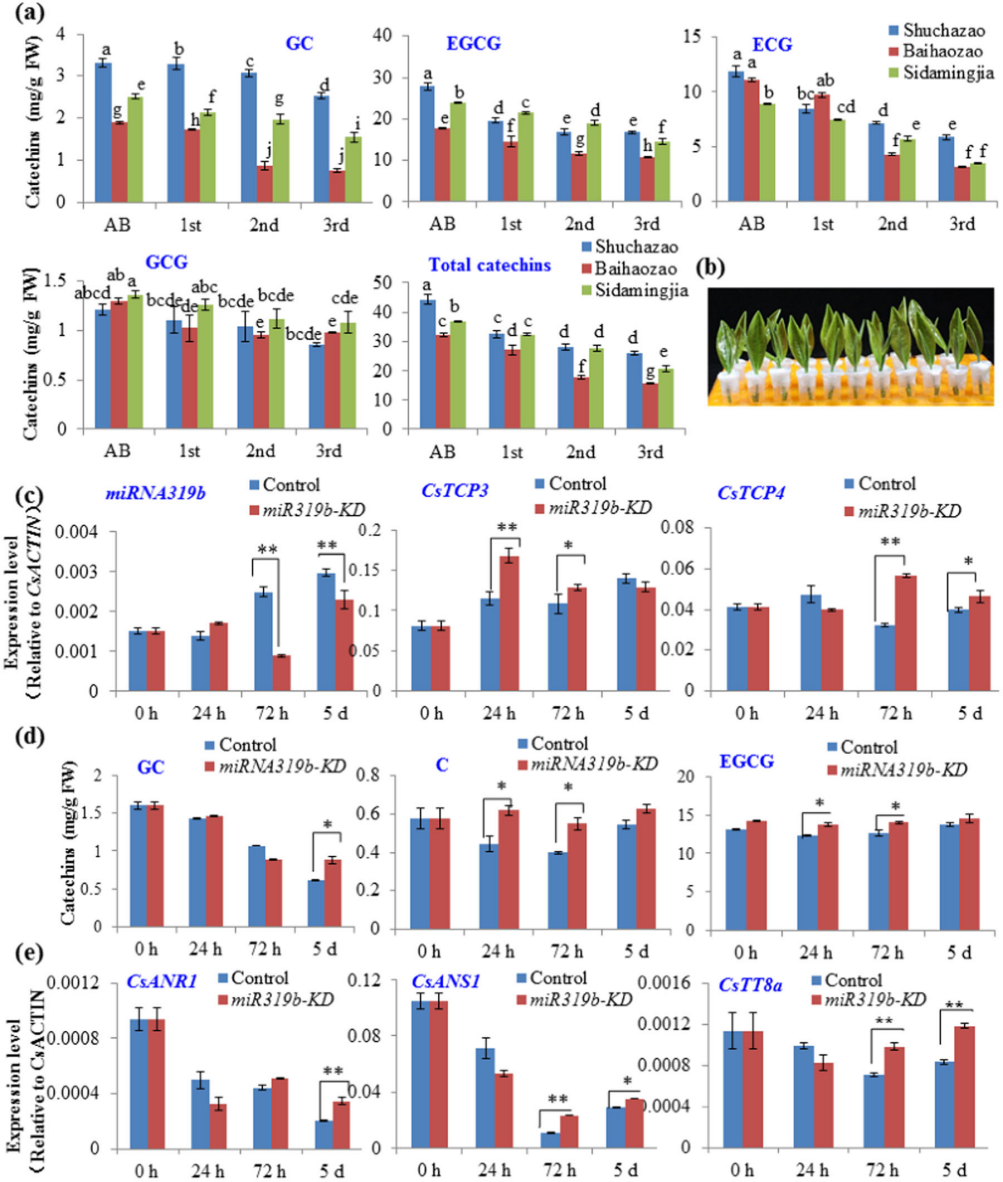
Involvement of *CsmiR319b/CsTCP* modules in catechin biosynthesis in tea plant leaves. **a** Contents of catechins in the apical bud (AB) and 1st, 2nd, and 3rd leaves of Shuchazao, Baihaozao, and Sidamingjia plants. Differences between tea plant varieties were analyzed via two-factor ANOVA using the LSD_0.05_ method. **b** Knockdown of *CsmiR319b* (*csmiR319b-*KD) with asODN and incubation, with a solution containing sense oligonucleotide serving as a control. **c** qRT-PCR verification of *CsmiR319b* knockdown (KD) and changes in the expression levels of *CsTCP3* and *CsTCP4* corresponding to treatment time. **d** Changes in catechin contents in *csmiR319b*- KD shoot tips treat ed with asOND. **e** Altered expression levels of *CsANR1*, *CsANS1*, and *CsTT8a* in *csmiR319b*-KD shoot tips treated with asOND. Significant differences between *csmiR319b*-KD and sODN controls were analyzed by using Student’s *t*-test in a two-tailed comparison (*P < 0.05 and **P < 0.01)

We further explored the relationship between *CsmicroR319b/CsTCP3-4* expression and catechin biosynthesis. The major catechins, such as EGCG, ECG, GC, and EGC, as well as total catechins decreased throughout leaf development. Among those of the three varieties, the leaves of Baihaozao had the lowest catechins, whereas catechin contents in the Shuchazao and Sidamingjia leaves were comparable (Fig. 8a).

Although no transgenic techniques have yet been developed specifically for tea plants, we knocked down the *CsmiR319b* transcript and monitored the effect on *CsTCP3* and -*4* expression and catechin levels by incubating the shoot tips in accordance with the antisense oligonucleotide (asODN) technique (Fig. 8b). Over longer incubation times, *CsmiR319b* transcript levels clearly decreased significantly (Fig. 8c). Knockdown of the *csmiR319b* transcript led to increased levels of *CsTCP3* and *CsTCP4* transcripts in the shoot tips after incubation, suggesting that the *CsmiR319b/CsTCP3-4* module can be manipulated (Fig. 8c). Interestingly, catechins in these treated samples also showed that *CsmiR319b* knockdown was accompanied by increased catechin content (Fig. 8d and Supplementary Fig. S11). Overall, this indicated an inverse relationship between *CsmiR319b* transcript levels and catechin accumulation. Further RT-PCR data showed that the expression of *CsANS1*, *CsANR1*, and *CsTT8a* was upregulated, indicating that it is likely that downregulation of *CsmiR319b* expression may upregulate *CsTCP3-4* expression, which then activates their target MBW-related and structural genes and ultimately alters the catechin content. Taken together, our results demonstrate that TCP TFs and their cognate microRNAs connect leaf and shoot development to catechin biosynthesis in the phenylpropanoid pathway in tea plant (*C. sinensi*s), opening a new research avenue for improving tea productivity in terms of both yield and quality.

## Discussion

Plant-specific TCP TFs play various roles in plant organ growth and development, cell proliferation, and biosynthesis of flavonoids^6,7,28,29^. Apical buds and young leaves are the major parts of tea plants used for making various types of teas, such as green, white, and black teas. The shape of the apical buds and young leaves together with their yields is one of the major factors affecting tea production. The accumulation of characteristic secondary metabolites such as catechins, theanine, and caffeine in the apical buds and young leaves is critical for tea quality^1^. Nevertheless, the molecular connections between shoot development and the accumulation of these secondary metabolites in tea plants remain unclear. By combining transcriptome and metabolite profiling with leaf morphology, miRNA expression analysis, and biochemical assays, our studies on CsTCP family TFs revealed some of their roles in determining tea plant leaf shape and regulating the biosynthesis of catechins.

### Roles of CsTCPs in the determination of tea plant leaf and shoot development

**Fig. 9:**
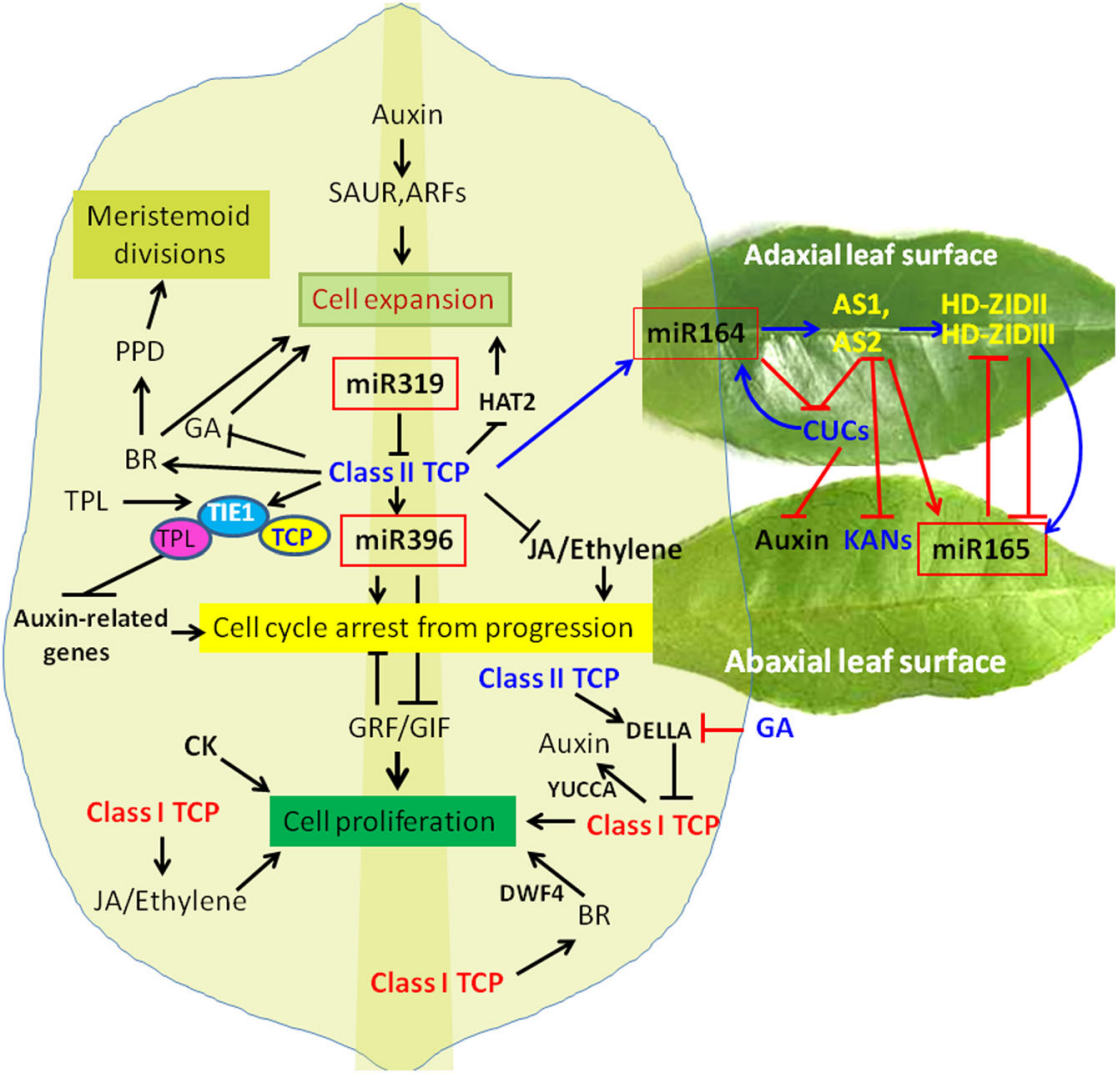
Proposed model for involvement of CsTCPs, miRNA/target modules, and other genes in tea plant leaf development. TOPLESS (TPL), TCP interactor containing EAR motif protein 1 (TIE1), PEAPOD (PPD), auxin response factor (ARF), Dwarf 4 (DWF4), Brassinosteroid (BR)

Like in other plant species, the development of tea plant shoot tips from the apical meristem is regulated by TFs in a spatiotemporal manner and is an integration of both endogenous hormone signals and environmental factors. These conserved regulatory mechanisms that precisely control leaf initiation, polarity establishment and maintenance, leaf flattening, and intercalary growth have been extensively studied^6,7,36^. Leaves initiate from the primordia on the ends of the SAM, and the leaf primordia are composed of adaxial and abaxial tissues that give rise to future upper and lower tissues of leaves (Fig. 9). The transcriptional regulatory networks controlling leaf and shoot development include many components: WUSCHEL (WUS)/CLAVATA (CLV) regulatory loop components; the auxin-, CK-, and GA-controlled KNOX regulatory module for meristem maintenance; and the HD-ZIPIII/AS1/2-KANADI (KAN)/ASYMMETRIC LEAVES 1/ROUGHSHEATH 2/PHANTASTICA (ARP) module for leaf adaxial/abaxial polarity specification and maintenance^6,7,36^. TCPs play critical and multifaceted roles in leaf growth and shape determination. Two cellular processes, division, and expansion, that are spatially and temporally coordinated in leaf morphogenetic development have been recognized^51,52^ (Fig. 9). Of them, CIN-type TCPs are key regulators of the timing of the transition from division to expansion within eudicot leaves. The expression and activity patterns of these TCPs modulate phytohormone responses during the spatiotemporal control of the cell fate transition through transactivation of cell cycle regulators, growth-repressing miRNAs, and/or interactions with chromatin remodeling machinery^6,7,16–18^. The profiling of more than 40 developmental-related genes essentially proved their high expression levels in developing tea plant buds and leaves (Figs. 2 and 5). Tea plant leaf shape should also be the outcome of the combined actions of multiple regulatory networks on the early development of leaf primordia (Fig. 9). WUS-like homeobox (WOX) TFs such as STENOFOLIA (STF) and WOX9 were shown to be critical regulators of leaf outgrowth in a regulatory network connecting AS and TCP^50–52^. NGATHA (NGA) TFs mediate the functions of AtTCP2 and AtTCP3 in leaf development^53^. NGA and STYLISH family gene transcripts are abundant in leaf margins, perhaps regulating leaf marginal shapes such as smooth, serrated (toothed), or lobed ones^54^. Thus, it is proposed that members of multiple TF families and hormones involved in the regulatory network specifically control tea plant leaf development (Fig. 9).

Class-I TCPs in Arabidopsis play positive roles in leaf growth and cell proliferation. AtTCP7 and AtTCP23 are involved in cell proliferation^39^. Correspondingly, CsTCP19 was reported to regulate leaf senescence in a manner that is redundant with CsTCP20 in *C. sinensi*s, similar to that in Arabidopsis^55^. It was proposed that class-I and class-II TCP TFs usually have antagonistic functions^17^ in the regulation of senescence and leaf development associated with JA biosynthesis and cell proliferation^6,7,55,56^. Class-I and class-II TCP TFs could antagonistically regulate the same target, balancing the regulation of development, growth, and other physiological functions or cellular processes, similar to the concept of Yin-Yang (Fig. 1). For example, in JA biosynthesis, class-I and class-II TCPs regulate the same target gene, *LIPOXYGENASE 2* (*AtLOX2*), by binding to different promoter elements^56^. AtTCP20 regulates leaf development by modifying LOX2. LOX2 is also targeted by AtTCP3, -4, and -10 under the control of *miR319*, and the manner of this targeting is antagonistic to that of AtTCP20^56^. Both the growth- and senescence-promoting effects of class-I TCPs are opposite those of the known class-II TCP mutants in *JAW* plants. The antagonistic regulatory effects of class-I and class-II TCP TFs on leaf development are likely to be mediated via the JA signaling pathway^57^.

### Involvement of *CsmiRNA/CsTCP* modules in the regulation of leaf development

Plants with altered *miRNA* expression have pleiotropic developmental defects^20–22,35^. The *JAW* locus encoding *miR319* represses *AtTCP3*, *-4*, and *-10* gene expression and controls leaf development; normal expression of *miR319* is required to prevent aberrant activity of AtTCP4 in leaf development^23^. For instance, high levels of *miR319* downregulate these *TCP* transcript levels and alter Arabidopsis leaf morphogenesis, leading to the production of crinkled leaves^18,22,35^. In addition to *miR319/CIN-TCP*s, the *miR396/HD-ZIPIII*s, *miR164b/CUC*s, and *miR396b*/*GRF-GIF* modules also critically regulate leaf development by modulating the differentiation or dedifferentiation balance in the shoot meristem or leaf cells^33,36^. This downregulation of *CIN*-*TCP* expression delays the arrest of cell proliferation within the margins and distal ends of leaves and petals, resulting in overproduction of cells in these regions^16,22,36^. Thus, downregulation of *CIN-TCP*s or overexpression of *miR319* caused curled, crinkled, or rolled leaves due to overgrowth and cell proliferation in certain leaf areas^22,36^. We also observed higher expression levels of *CsmiR319b* but lower levels of *CsTCP3* and -*4* in Sidamingjia and, to a lesser extent, in Baihaozao, corresponding to crinkled or rolled leaves (Fig. 6). The Arabidopsis *tcp2tcp4* double mutant has enlarged flat leaves, and the *tcp2tcp3tcp4tcp10* mutant has highly crinkled leaves due to delayed differentiation, upregulation of cyclin and other cell division gene expression, and extended mitotic activity in the marginal regions of the leaves^54^. *miR319*-mediated repression of *CIN-TCP* genes is required for normal organ development. The *CsTCP2*, *-3*, *-4*, or *-10* genes also have complementary *miR319* recognition sites, indicating that the expression of these genes could also be regulated by *miR319* and affect leaf development in tea plants.

The *miR164/CUC* module regulates leaf margin dissection^18,36^. *AtTCP3* directly activates the expression of *miR164*, *AS1*, *INDOLE-3-ACETIC ACID 3*/*SHORT HYPOCOTYL 2*, and *SMALL AUXIN UP RNA* (*SAUR*) genes^6,7,36^. In Arabidopsis, redundant but partially distinct functions of CUC1, CUC2, and CUC3 control the formation of embryonic shoot meristems and boundaries between meristems and emerging organ primordia throughout the plant life cycle^46^. *miR164* negatively regulates *AtCUC1* and *AtCUC2*, whose mutants fail to establish organ boundaries and show severe developmental defects^18^. Compared with those of Shuchazao, developing leaves of both Sidamingjia and Baihaozao had higher expression levels of *CsCUC* genes, corresponding to their curved and extended length, as well as longer internodes (Fig. 6). By contrast, overexpression of *AtTCP3* activates *miR164* and suppresses *AtCUC* gene expression, resulting in the fusion of cotyledons and formation of defective shoot meristems^18^. These CIN-like *TCP* genes thus act redundantly in leaf differentiation in a dose-dependent manner, and their diversity has important roles in the signaling pathways that generate different leaf forms^6,7,18,36^.

The *miR396/GRF-GIF* regulatory module also plays crucial roles in controlling the development of plant tissues and organs^6,30,45^. The HD-ZIPII protein HAT3 physically interacts with HD-ZIPIII proteins and directly represses *miR165/166* expression^31^. AtTCP4 imparts differentiation competence by promoting the auxin response and activating HD-ZIP II HAT2 downstream of the auxin response to restrict the cell number and final size of leaves^6,7^. Most tea plant leaves had similar sawtooth margins; thus, the *miR396/CsPHV-CsREV1a* modules in these varieties did not show drastic changes. Compared with those of the third variety, crinkled leaves of both Shuchazao and Sidamingjia had higher expression levels of *CsGRF1* (Fig. 6).

A previous transcriptome study implied that *miR319c/CsTCP2* regulates apical bud burst in tea plants^47^, and other *miRNA*s play roles in tea plant bud dormancy and hormone responses^58,59^. All these data, together with those of our study, strongly indicate the essential roles of *miRNA*s in the regulation of tea plant shoot tip activities and leaf development.

### *CsTCPs* are involved in regulating tea plant secondary metabolism

The correlation analysis of metabolite-*CsTCP* gene expression in tea plant tissues of different developmental stages and under light/shade conditions supported the close relationships between *CsTCP* genes and catechin production in tea plants. CIN-type TCPs and some PCF-type TCPs were closely related to catechin biosynthesis. However, our study further demonstrated that CsTCP3 and CsTCP4 physically interacted with CsTT8 and even with CsTT2, CsTTG1, and CsMYB75. A bHLH domain-containing TF, CsTCP3, interacted with CsTT8 to form a heterodimer. A PCF-type CsTCP14 also interacted with CsTT8, which is probably similar to CsTCP3. This is consistent with the fact that TT8s in various plant species form homodimers, which are assumed to bridge the MBW interaction complexes^44,60^. The conserved Cys in AtTCP15 or AtTCP14 leads to inhibition of DNA binding when oxidized and thereby inhibits anthocyanin accumulation during exposure to high-light stress^28^. These genes affect the expression of *PAP1/2* and *TT8* and anthocyanin biosynthesis-related genes in Arabidopsis^28^. Overexpression of *AtTCP3* significantly promotes the transcriptional activation complex of MBWs by interacting with TT8 to form a heterodimer^29^. MBW ternary complexes were also confirmed in tea plants to have regulatory functions in both anthocyanin and catechin biosynthesis^3,4^. CsTT2, CsMYB75, CsAN1, CsTT8, and CsTTG1 were identified as components of MBW complexes that activate flavonoid biosynthesis-related genes such as *CsANS1* and *CsANR1*. Our study showed that CsTCP3 and CsTCP14 modified the MBW complex by interacting with their components and by strengthening or interfering with the activity of those complexes. This has further expanded the concept that CsTCP3 interacts with CsTT8 and CsTT2 but not with the repressor CsMYBL2. Our study indicated that CsTCP3, CsTCP4, or, likely, CsTCP14 could form homodimers by themselves and heterodimers with CsTT8a. Furthermore, both CsTCP3 and CsTCP14 acted through modification of the activity of MBW complexes via interactions with the components of those complexes. Interestingly, the Medicago leaf development regulator STF also activated flavonoid biosynthesis and MtTCP3^50,52^.

Apparently, the catechin content in tea plant leaves is a quantitative trait that is determined by not only more than 30 structural genes but also a number of endogenous regulatory factors, as well as environmental cues^1^. CsTCP3/4 and CsTCP14 are transcriptional regulators that can mediate endogenous signals, such as hormones, or environmental factors, such as light, to alter catechin biosynthesis in young tea plant leaves. As reported previously, TCP14 mediates the alteration of anthocyanins in response to high light^28^, and CsTCPs may be involved in the regulation of catechin levels in different tea plant varieties under different environmental conditions. Such functional variations in these CsTCPs with regard to tea plant varietal differences based on different locations and environments will need further validation under multienvironmental conditions.

### Conclusions

We identified 35 *CsTCP* TFs from the tea plant genome, and two classes of *CsTCP* TFs were expressed in tea plant tissues and organs to varying degrees, with expression patterns similar to those of their counterparts in Arabidopsis with conserved functions. We demonstrated that CsTCP3 and CsTCP4 are, at a minimum, TFs that can integrate the regulation of shoot tip and leaf developmental processes together with the biosynthesis of catechins in tea plants. Furthermore, our study revealed the regulatory modules of various sets of *CsmiRNA*/target *CsTCP*s or other regulatory genes, such as *CsmiR319/CIN-CsTCP*s, *CsmiR396/CsGRFs/GIF*s, *CsmiR164/CsCUC*, and *CsmiR165*/*CsPHB-CsPHV*, involved in tea plant leaf development. Moreover, the *CsmiR319b/CsTCP3-4* modules are correlated not only with leaf development but also with catechin biosynthesis. These results were shown by using biochemical assays to form regulatory complexes together with MBWs that functionally regulate target metabolic gene promoters and by knocking down *CsmiR319b* in shoot tips for effective control of *CsTCP3/4* expression and catechin accumulation. This study also revealed that TCPs are among the important regulators involved in the complex regulation of tea plant shoot tips and leaf development and demonstrated that they coordinate the regulation of leaf morphology with catechin biosynthesis in the developing leaves of tea plants in response to endogenous and environmental signals.

## Materials and methods

### Plant materials, growth conditions, and experimental treatments

Twenty-five-year-old tea plants produced by cuttings in the town of Dayang (31°55′ north, 117°12′ east; Hefei, Anhui Province, China) were used for phenotyping leaf shape, stem branching, and shoot tip development. The following tissues types were sampled during the spring and summer at 20–27 °C under a 12 h/12 h light/dark photoperiod: shoot tips; apical buds; 1st, 2nd, 3rd, 4th, and 5th leaves; and 1st, 2nd, 3rd, and 4th internodes of the stems. The apical buds, leaves, and stems were sampled from at least nine individual tea plants of three different tea plant (*C. sinensis*) varieties, Shuchazao, Baihaozao, and Sidamingjia, which were bulked into three pools as biological repeats. A similar sampling method was used for the treatment experiments. Each treatment was replicated three times. These materials were used for metabolite analysis, RNA extraction for transcriptome sequencing or qRT-PCR analysis. Transcriptomic data from various experimental treatments (MeJA, polyethylene glycol, NaCl and cold, and shade) were retrieved from previous studies and the Tea Plant Information Archive (http://tpia.teaplant.org/index.html)^43,61–63^.

### RNA extraction, transcriptome sequencing, and quantitative real-time polymerase chain reaction (qRT-PCR) analysis

For transcriptome analysis, total RNA was isolated from tissues using an RNAprep Pure Plant Kit and treated with DNase I (Tiangen; http://www.tiangen.com). The RNA concentration and quality were assessed using a Thermo 2000 Bioanalyzer and an RNA NanoDrop ND-2000 Spectrophotometer (Thermo Fisher Scientific, Co., Ltd., Shanghai, China). The purified RNA was reverse transcribed to cDNA and then sequenced on an Illumina HiSeq 2500 platform by BGI Shenzhen Biotechnology Company according to routine processes as described previously^64^. The reads per kilobase per million mapped reads (RPKM) and read counts were calculated using eXpress.

For miRNA analysis, total RNA was extracted from tea plant tissues using a TRIzol kit (Transgen Biotechnology Co., Ltd., Beijing, China) and reverse transcribed into cDNA using a PrimeScript™ RT Reagent Kit together with gDNA Eraser (Takara Biotechnology Co., Ltd., Dalian, China) according to the manufacturer’s instructions. Information about the mature microRNAs in tea plant buds and young leaf tissues, including miR319s, miR164s, miR396s, and miR165/166s, was obtained from previous microRNA sequencing results. These mature miRNAs were reverse transcribed and measured using a PrimeScript™ RT Reagent Kit (Perfect Real Time) (Takara Biotechnology Co., Ltd., Dalian, China). For miRNA, 0.5 μg of total RNA in a 10-μL volume, 2 μL of 5× PrimeScript buffer, 0.5 μL of PrimeScript RT Enzyme Mix I, 0.5 μL of miRNA-RT primer, 0.5 μL of random hexamers, and RNase-free dH_2_O were used. The PCR conditions were 42 °C for 60 min followed by 95 °C for 3 min, after which dilution to 150 ng/μL with water was performed. A SYBR Premix Ex Taq Kit (Takara Biotechnology Dalian Co., Ltd., Dalian, China) was used for qRT-PCR on a Bio-Rad iQ5 fluorescence quantitative PCR platform. Five microliters of qRT-PCR product was subjected to 2% agarose gel electrophoresis. *CsmiR222* was used as an internal reference for normalization of miRNA expression levels. The primers used for qRT-PCR of miRNAs are listed in Supplementary Table S1.

For qRT-PCR, *CsACTIN* was used as the reference for normalization using the primer pairs listed in Supplementary Table S1. A SYBR Premix Ex Taq kit was used for qRT-PCR on a Bio-Rad iQ5 fluorescence quantitative PCR platform according to a previously described method^65^.

### Identification of CsTCP family genes in the tea plant genome

The sequences of the TCP proteins of Arabidopsis and rice were retrieved from the TAIR (https://www.arabidopsis.org/) and rice genome databases (http://rice.plantbiology.msu.edu/), respectively. The sequences from Arabidopsis were subjected to a multiple-database search against the tea plant genome sequence, which was downloaded from the Tea Plant Information Archive (http://tpia.teaplant.org/index.html). The amino acid sequences were aligned using ClustalW, and MEGA 6.0 software was used to construct a phylogenetic tree by the NJ method, with 1000 bootstrap replicates. The ExPASy proteomics server (http://expasy.org/) was used to predict the isoelectric point and molecular weight of the CsTCP proteins. The exon/intron structures of individual CsTCP genes were defined by comparing the coding sequences and corresponding genomic sequences via the Gene Structure Display Server (http://gsds1.cbi.pku.edu.cn/), and the conserved motifs were analyzed using the MEME program (http://meme-suite.org/). PlantCARE (http://bioinformatics.psb.ugent.be/webtools/plantcare/html/) was used to determine the distribution of putative *cis*-acting elements in the 1400-bp promoter sequence of 35 CsTCPs. The Pfam (http://pfam.xfam.org) and SMART (http://smart.embl-heidelberg.de) tools were subsequently used to identify conserved TCP domains and R domains. The identification of miRNA-targeted genes was performed according to a previously described method^47^. To visualize the protein domain structures, IBS 1.0 software (http://www.mybiosoftware.com/ibs-illustrator-of-biological-sequences.html) was used.

### Correlations of gene expression and metabolite accumulation

The transcriptomic data and metabolite data from 8 or 12 different tissues of the tea varieties Shuchazao and Longjin 43 were downloaded from the Tea Plant Information Archive (http://tpia.teaplant.org/index.html). The transcript levels of the *CsTCP* genes in each tissue were calculated using the log10(FPKM) value, after which R software was used to visualize the CsTCP expression patterns. The transcriptomic data concerning the expression level in ten tea plant tissues (first, second, third, fourth, and fifth leaves; old leaves; and first, second, third, and fourth internodes of the stems) were used for correlation analysis according to the method above. Metabolite profiling was performed by the use of high-performance liquid chromatography (HPLC)^64^. To identify TCPs associated with flavonoids, caffeine, and theanine, correlation analysis between the *CsTCP* genes and these metabolites was performed by the use of Pearson’s correlation coefficients. Correlations whose correlation coefficient (*r*) was >0.5 and whose *p*-value was <0.05 were considered statistically significant. In the representative figure, blue color means a positive correlation, and red color means a negative correlation. Transcriptome and metabolic profiling data sets were obtained from different tissues of tea plants^64^.

### Yeast two-hybrid assays and BiFC assays

The ORFs of *CsTCP3* (GenBank accession No. MW071231), *CsTCP4* (GenBank accession No. MW071232), *CsTCP14* (GenBank accession No. MW071233), *CsTT8a*, *CsTT2a*, *CsMYBL2*, *CsMYB75*, and *CsTTG1* were cloned via pairs of gene-specific primers that have specific restriction sites (Supplementary Table S1). Protein–protein interaction assays were conducted according to previously described methods^44^. For subcellular localization assays, the ORFs of *CsTCP3* and *CsTT8a* were cloned and inserted into pCAMBIA1300 (CAMBIA, Canberra, Australia) vectors by the in-fusion technique. The constructs were subsequently fused to the N-terminus of mCherry, after which the transformants were transformed into *Agrobacterium tumefaciens* strain GV3101. *Nicotiana benthamiana* leaves were infiltrated with agrobacteria harboring the constructs for transient expression of fusion proteins as described previously^66^.

For BiFC assays, *CsTCP3* and *CsTT8a* ORFs were amplified and subcloned into pCAMBIA1300-eYFPN (the YFP N-terminal region) and pCAMBIA1300-eYFPC (the YFP C-terminal region) (CAMBIA, Canberra, Australia) vectors according to the in-fusion technique^66^. The resulting constructs were then transformed into *A. tumefaciens* strain GV3101, the cells of which were infiltrated into *N. benthamiana* leaves separately or in different combinations. A Leica DMi8 M laser scanning confocal microscopy system (Leica Microsystems, Wetzlar, Germany) was used for fluorescence observations, according to previously described methods^66^.

### Transactivation assays of *CsANS1* and *CsANR1* promoters

Genomic sequences that were ~2 kb upstream of the translation start codon of both the *CsANR1* and *CsANS1* genes were amplified from *Camellia sinensis* genomic DNA by the use of primers (Supplementary Table S1), after which the sequences were subcloned into p2GWL7 (http://gateway.psb.ugent.be) vectors, resulting in promoter::luciferase reporter constructs. Similarly, effectors (*CsTT8a*, *CsMYB75*, *CsTTG1*, *CsTT2a*, *CsTCP3*, *CsTCP14*) were cloned into p2GW7 vectors by LR reactions to form 35S::effector constructs by the use of previously constructed vectors^44,66^. Promoter transactivation assays were conducted according to described previously methods^44,66^.

### Suppression of *CsmiR319b* in tea shoot tips

Since transformation techniques have not yet been developed specifically for tea plant, the knockdown of candidate genes with antisense oligonucleotides (asODNs) containing complementary segments of the target gene was performed to determine how *CsmiR319b* affects *CsTCP3* and -*4* expression and catechin production in tea shoot tips, according to previously described methods^65,67^. The antisense oligonucleotides for *CsmiR319b* were designed according to Soligo software (http://sfold.wadsworth.org/cgi-bin/soligo.pl), with the *CsmiR319b* sequence used as an input sequence (Supplementary Table S1). To silence *CsmiR319b* expression, freshly detached healthy apical buds and 1st leaves from at least 5 tea plants of the variety Baihaozao were incubated in 1.5 mL Eppendorf tubes that contained 33 μM asODN solution for various times. Shoot tips incubated in a 10 mM sucrose solution together with sense ODNs (sODNs) were used as controls. The shoot tips were sampled at different time intervals to analyze the RNA and catechin levels.

### Data analysis

The experiments were performed for at least three biological repeats. Statistical analysis was performed using either Student’s two-tailed *t*-test when comparing treatments with controls or multiple comparisons together with the ANOVA multiple range test at the 0.05 probability level (*p* < 0.05).

## Supplementary information

Supplementary Figures S1-S11

Supplementary dataset S1-S9

## Data Availability

The author responsible for the distribution of materials integral to the findings presented in this article in accordance with the policy described in the instructions for authors is Jian Zhao (jianzhao@ahau.edu.cn).
