# Supplementary material for: CsTCPs regulate shoot tip development and catechin biosynthesis in tea plant (Camellia sinensis)
Source: Hortic Res. 2021 May 1;8:104. doi: 10.1038/s41438-021-00538-7 (PMC8087681; doi:10.1038/s41438-021-00538-7)
Supplement: Supplementary file 1 — Supplementary Figures S1-S11 [file 41438_2021_538_MOESM1_ESM.pdf]

Supplemental Fig S1 :

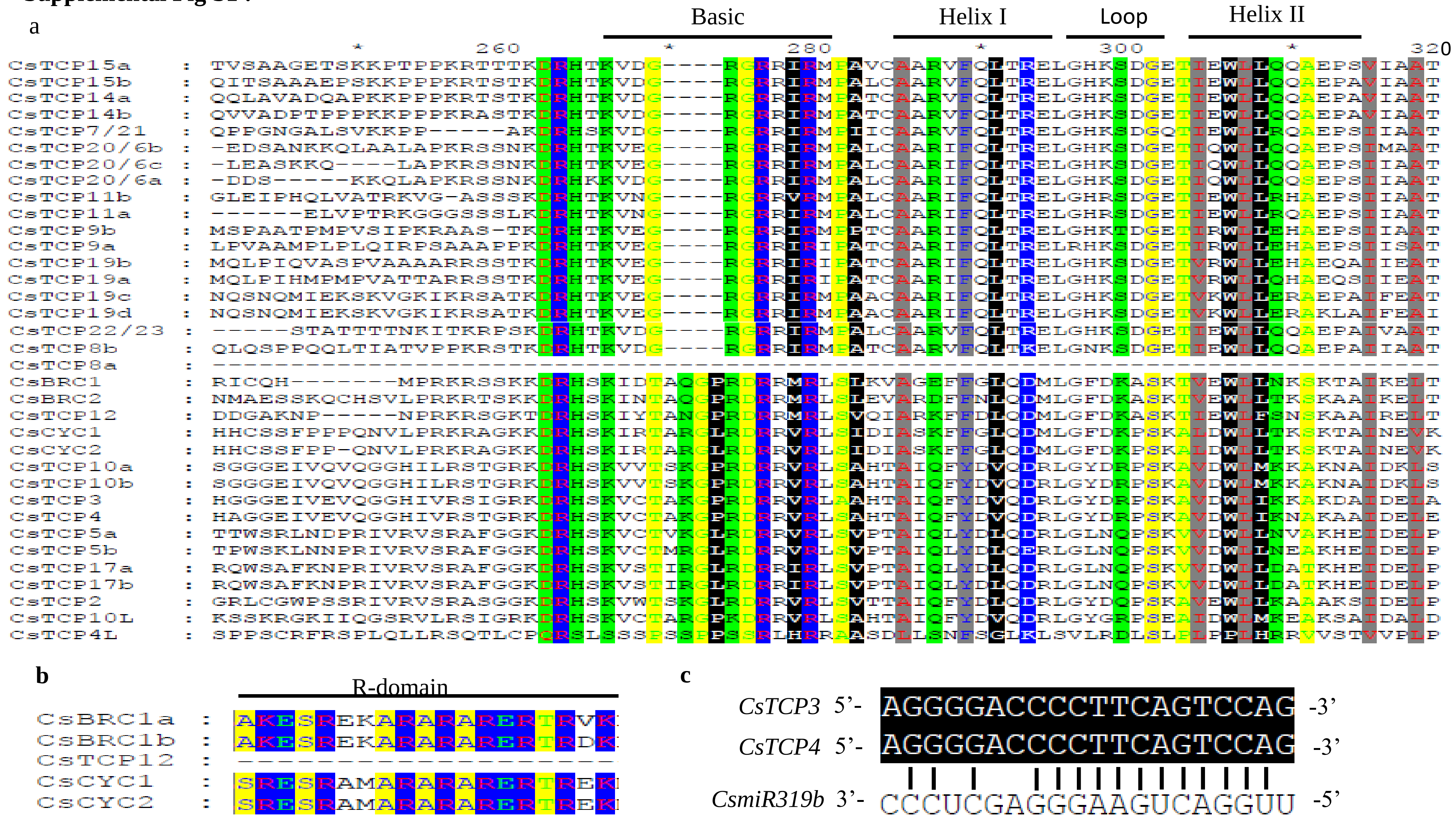

Supplemental Fig S1: Alignment of TCP domain of the 35 TCP proteins in *Camellia sinensis*.

(a) Alignment of TCP domain of the 35 TCP proteins in *Camellia sinensis* . Amino acids that are conserved throughout are shaded in different colors. Conserved domains, including Basic, Helix I, Loop, and Helix II, are shown at the top. (b) Alignment of R-domain of the CYC/TB1 subclass members. Conserved amino acids are shaded in different colors. Multiple sequence alignment was carried out using ClustalW and visualized with GenDoc software. (c) Alignment of CsmiR319b with its target genes CsTCP3 (922-941bp) and CsTCP4 (958-977bp) of cloned sequence from the shuchazao variety, with complementary sequences of CsmiR319b (1-20 nt).

Supplemental Fig S2:

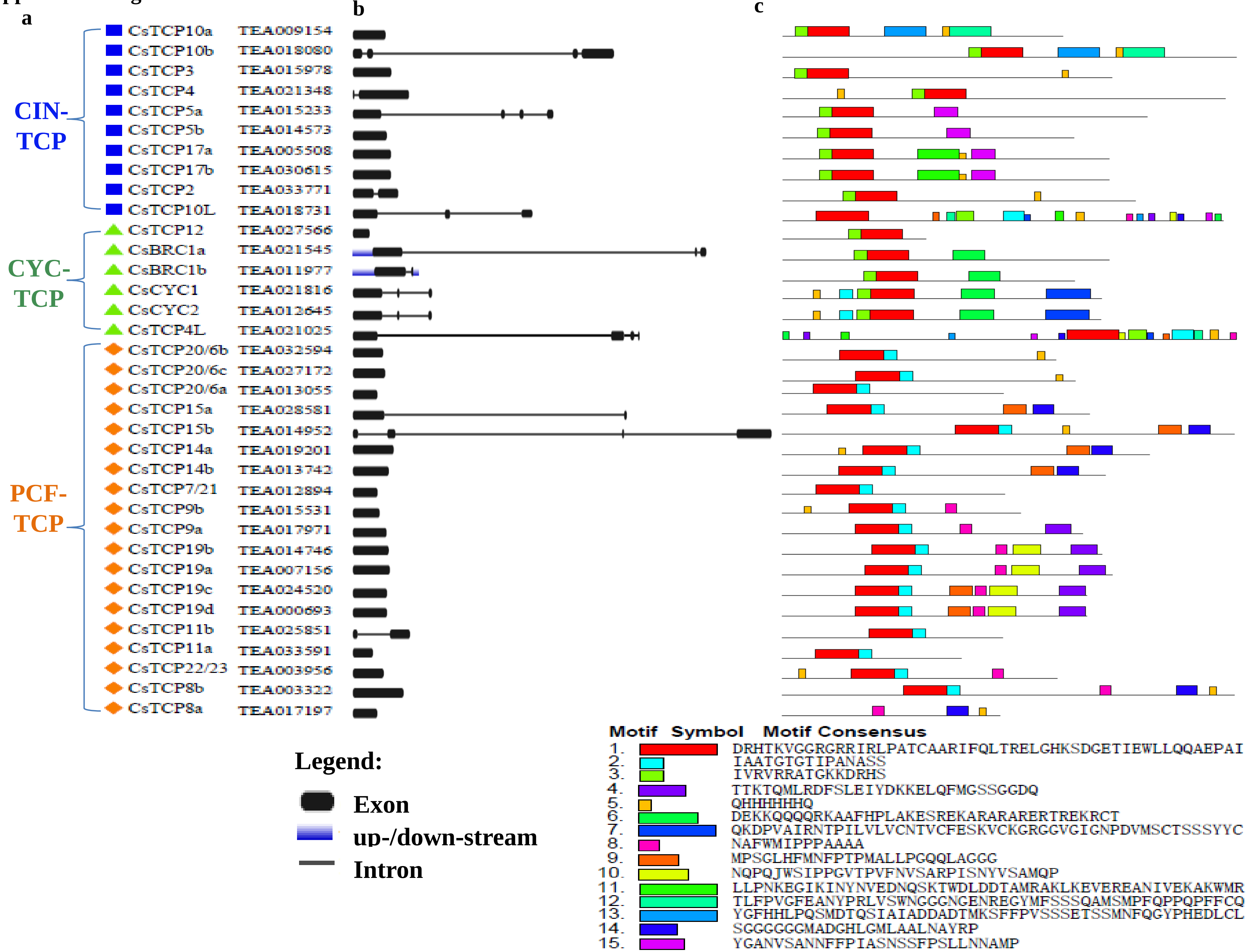

Supplemental Fig S2: The annotation and structural analysis of CsTCP genes in *Camellia sinensis*.

(a) Annotation of CsTCP genes. Phylogenetic analysis classified CsTCP genes into class I TCPs (PCF TCPs in brown color), and class II TCPs (including CIN-type TCPs in blue color, and CYC-type TCPs in green color). The gene TEA ID was given to each CsTCP gene.
(b) Analysis of *CsTCP* gene structures. Introns and exons were represented by a black line and box, respectively. The blue shows 3' or 5' UTR sequence.
(c) Analysis of the conserved motifs in CsTCP proteins. Different motifs were indicated by a colored box numbered from 1 to 15.

Supplemental Fig S3:

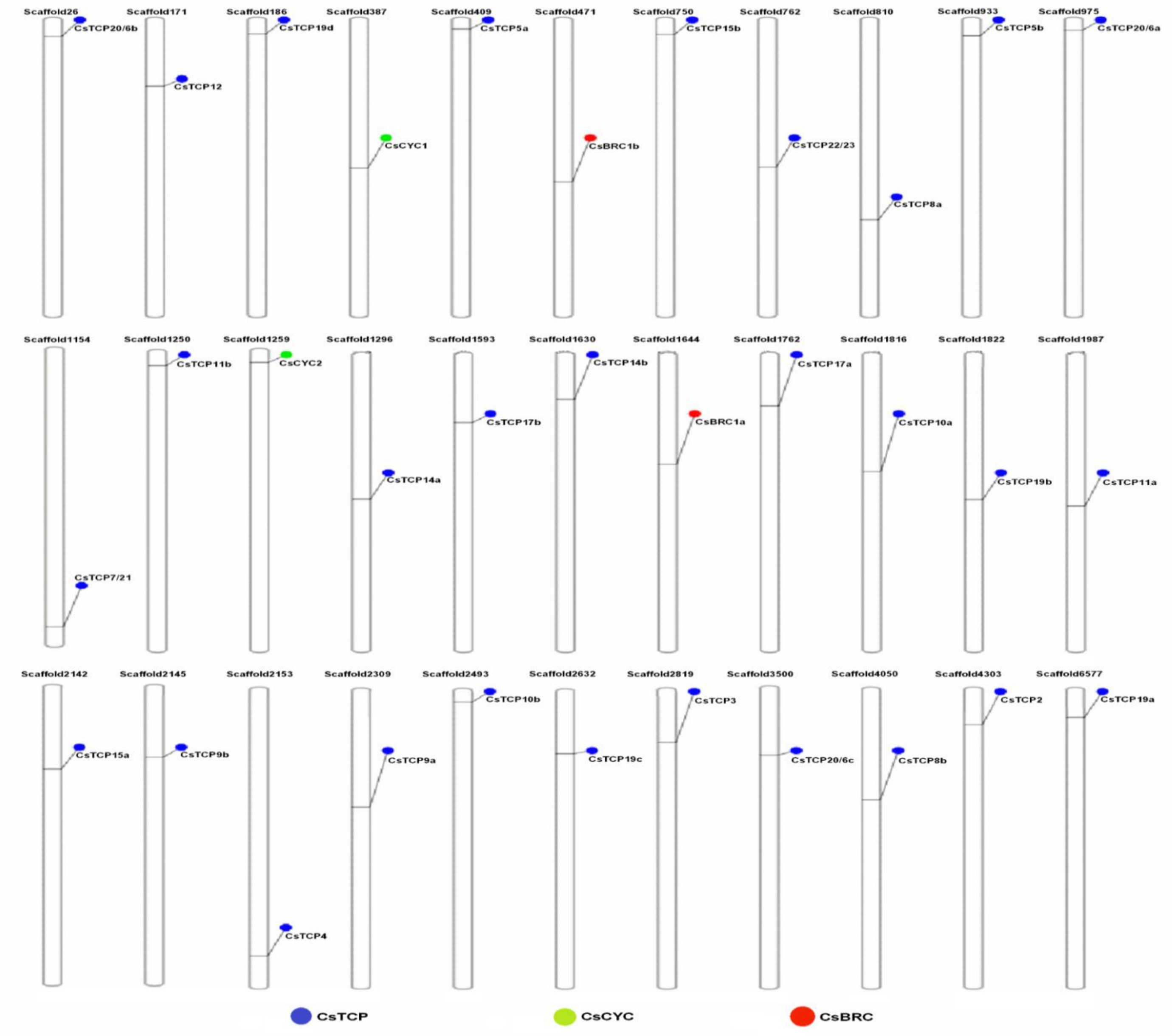

Supplemental Fig S3: The physical position of CsTCP genes in *Camellia sinensis*.
The scaffold ideogram was constructed on the basis of the physical position of CsTCP genes in *Camellia sinensis* . PhenoGram Plot (http://visualization.ritchielab.psu.edu/phenograms/plot; Wolfe et al., 2013) was used to create the image of CsTCP genes on different scaffold using the available information.

Supplemental Fig S4:

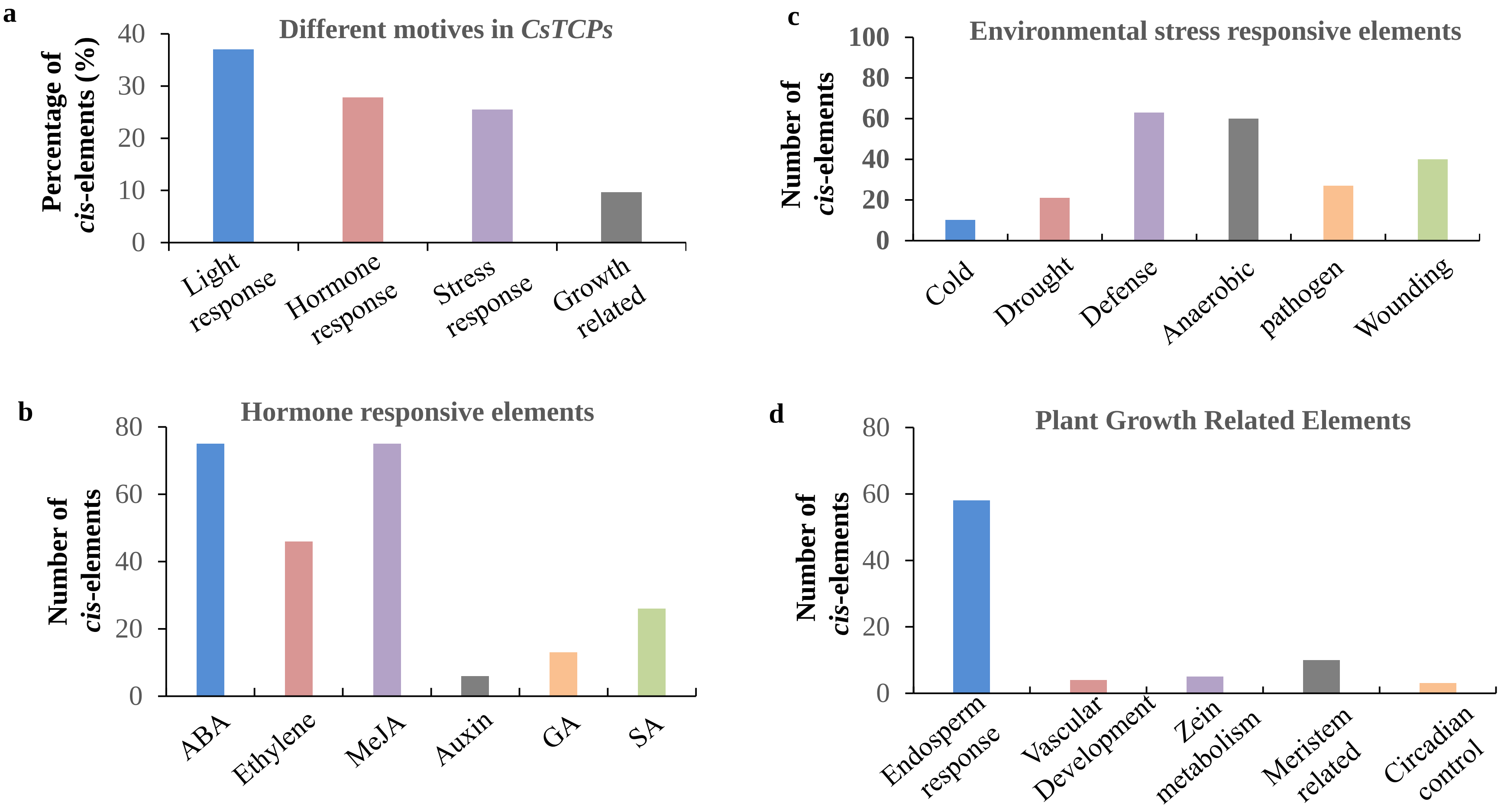

Supplemental Fig S4: Analysis of c*is* elements in the promoter regions of *CsTCP* genes in *Camellia sinensi*s.

(a) The percentage of *cis*-elements of CsTCP promoter regions. (b-d) Number of *cis*-elements in the promoter of CsTCP genes that are related to stress responses, hormone responsive plant growth and development.

Supplemental Fig S5:

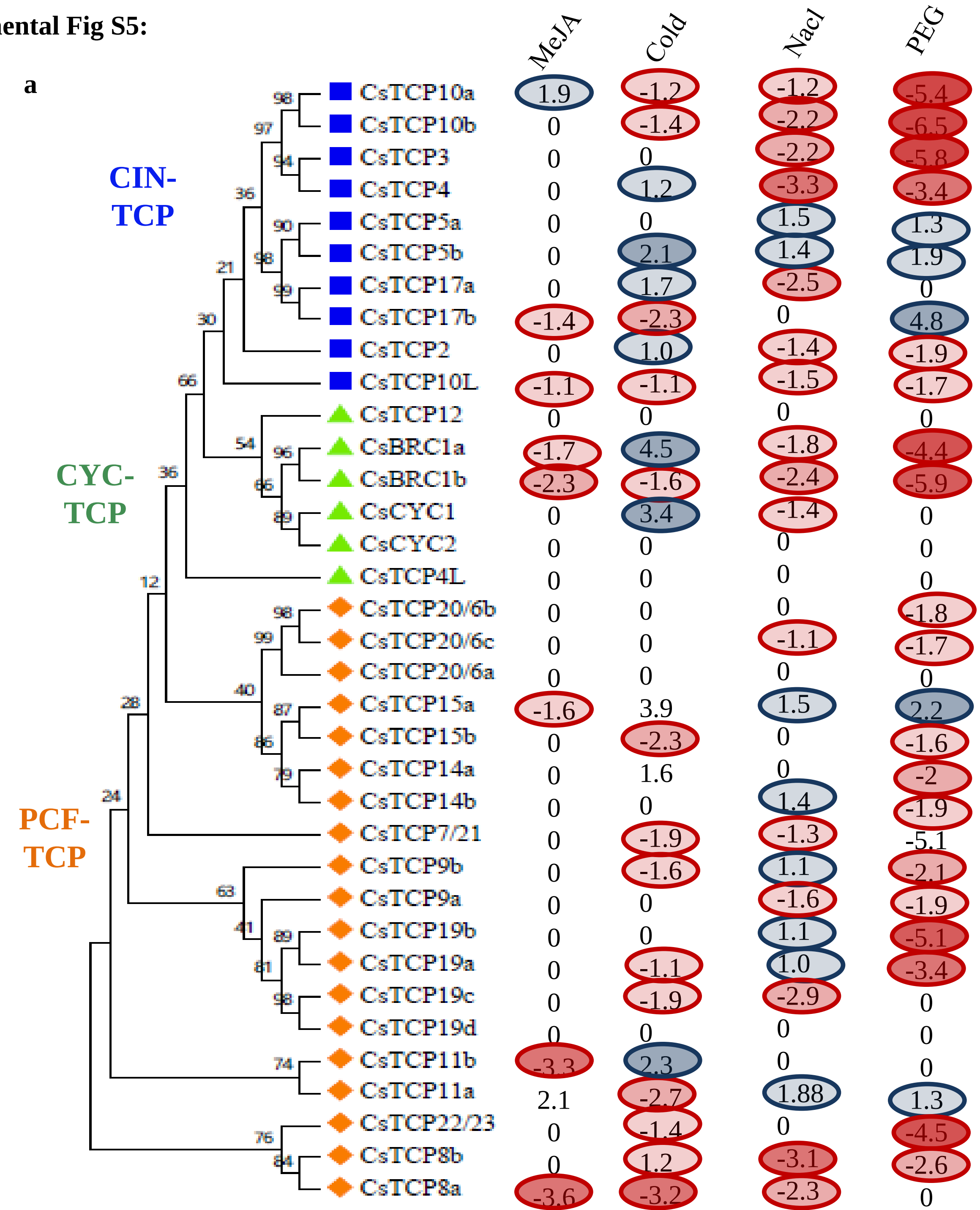

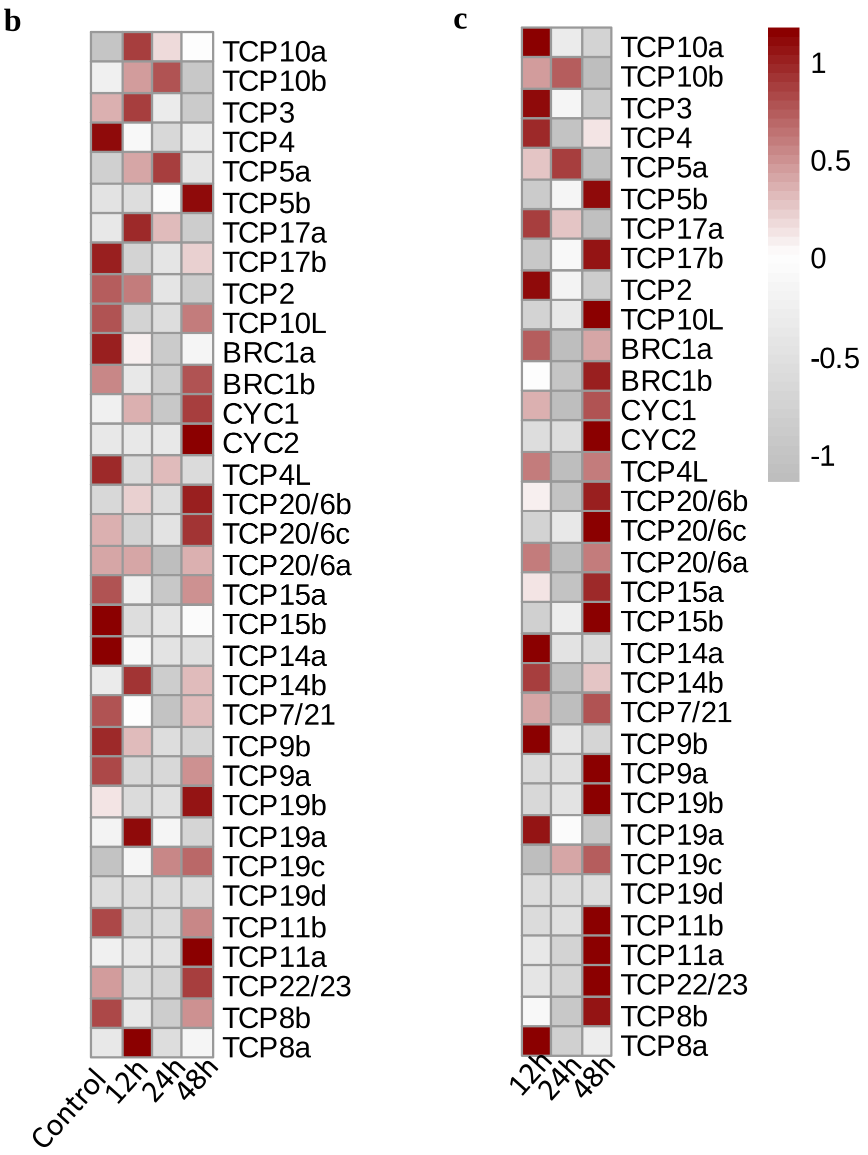

Supplemental Fig S5: Expression profiles of CsTCPs in tea plants treated with MeJA, cold, drought, and NaCl stresses.

(a) Expression pattern of CsTCPs *in response to MeJA and abiotic stress. Genevestigator analysis of CsTCPs using the Log2(Experiment/Control)* in response to MeJA, NaCl, drought and low temperature. (b) Expression patterns of *CsTCP*s in tea plants MeJA treatment. (c) Comparative expression of tea shoot tips under MeJA treatment. The ratio of treatment/ control was shown in the heatmap analysis.
Data were retrieved from previous publications (Shi et al., 2015; Zhang et al., 2017 ; Liu et al., 2018; http://tpia.teaplant.org/index.html)

Supplemental Fig S6:

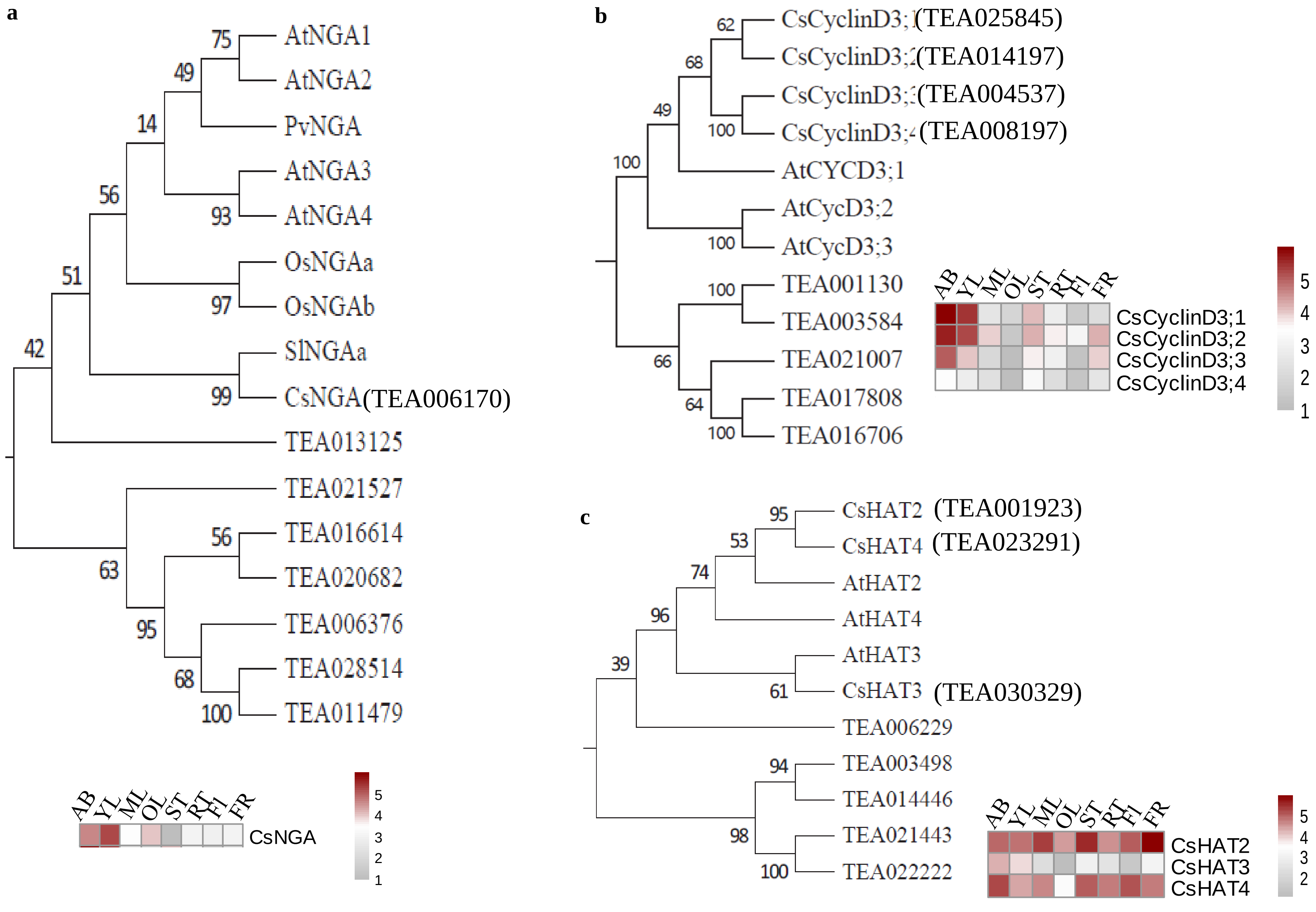

d

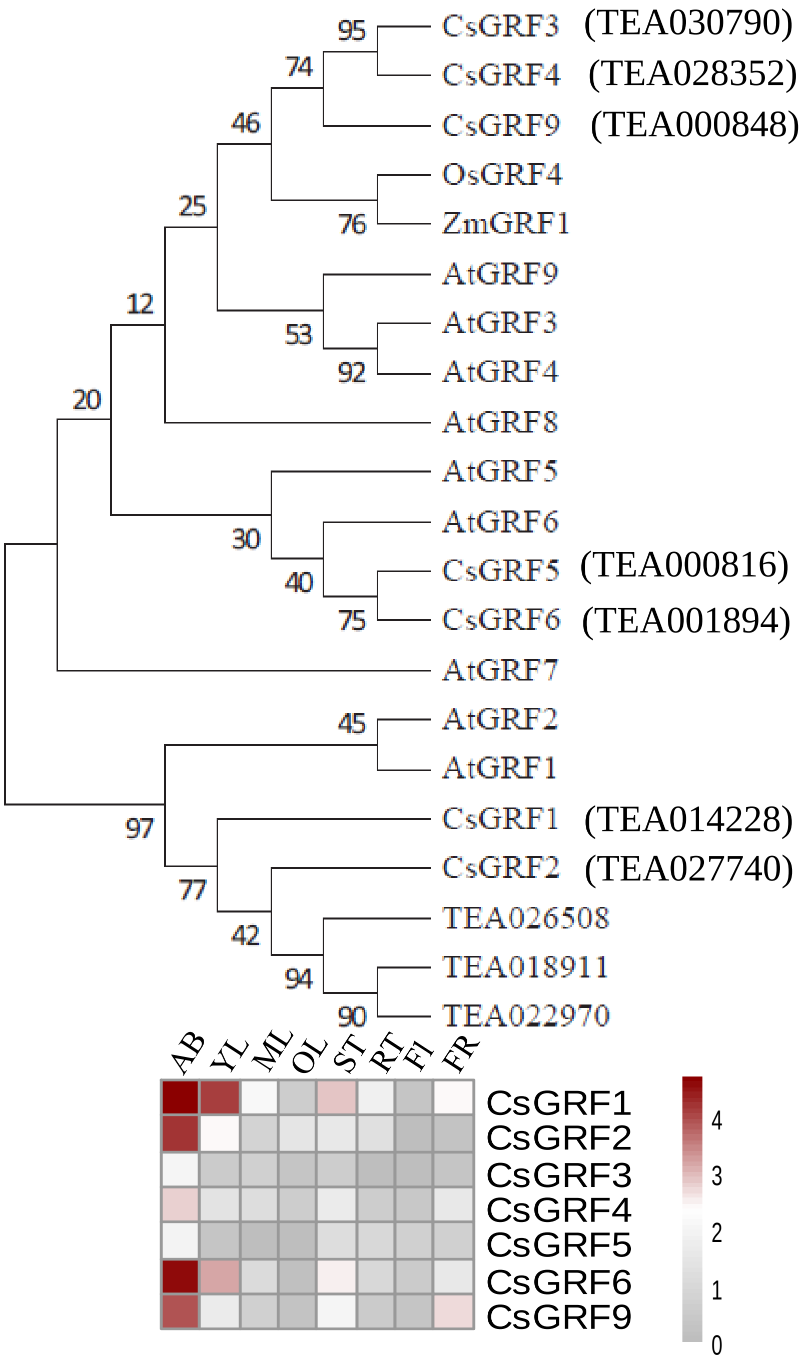
CsGRF3 (TEA030790)
CsGRF4 (TEA028352)
CsGRF9 (TEA000848)
OsGRF4
ZmGRF1
AtGRF9
AtGRF3
AtGRF4
AtGRF8
AtGRF5
AtGRF6
CsGRF5 (TEA000816)
CsGRF6 (TEA001894)
AtGRF7
AtGRF2
AtGRF1
CsGRF1 (TEA014228)
CsGRF2 (TEA027740)
TEA026508
TEA018911
TEA022970
95
74
46
76
25
53
92
12
20
30
40
75
45
97
77
42
94
90
AB
YL
ML
OL
ST
RT
Fl
FR
CsGRF1
CsGRF2
CsGRF3
CsGRF4
CsGRF5
CsGRF6
CsGRF9
4
3
2
1
0

e

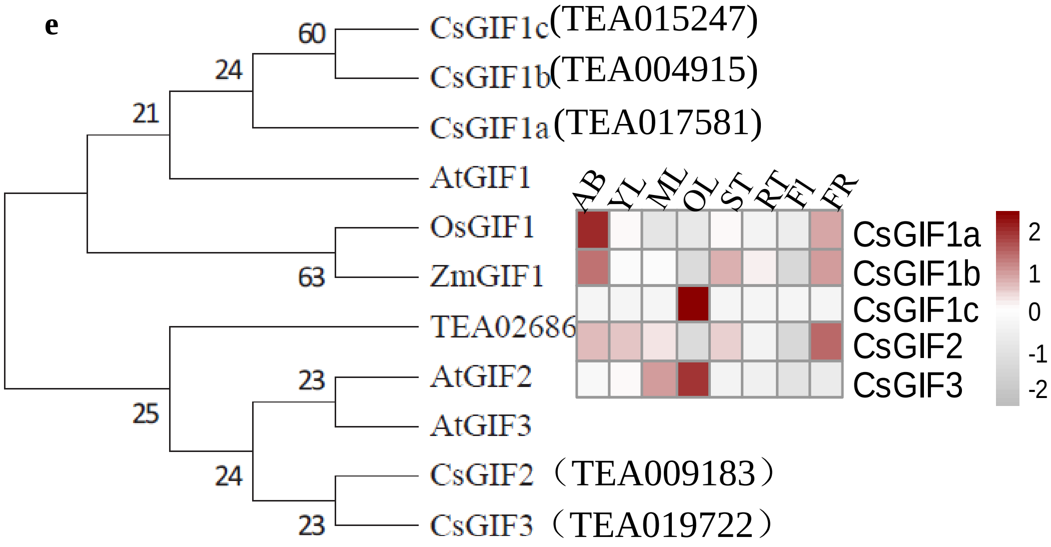
CsGIF1c(TEA015247)
CsGIF1b(TEA004915)
CsGIF1a(TEA017581)
AtGIF1
OsGIF1
ZmGIF1
TEA02686
AtGIF2
AtGIF3
CsGIF2（TEA009183）
CsGIF3（TEA019722）
60
24
21
63
25
23
24
23
AB
YL
ML
OL
ST
RT
Fl
FR
CsGIF1a
CsGIF1b
CsGIF1c
CsGIF2
CsGIF3
2
1
0
-1
-2

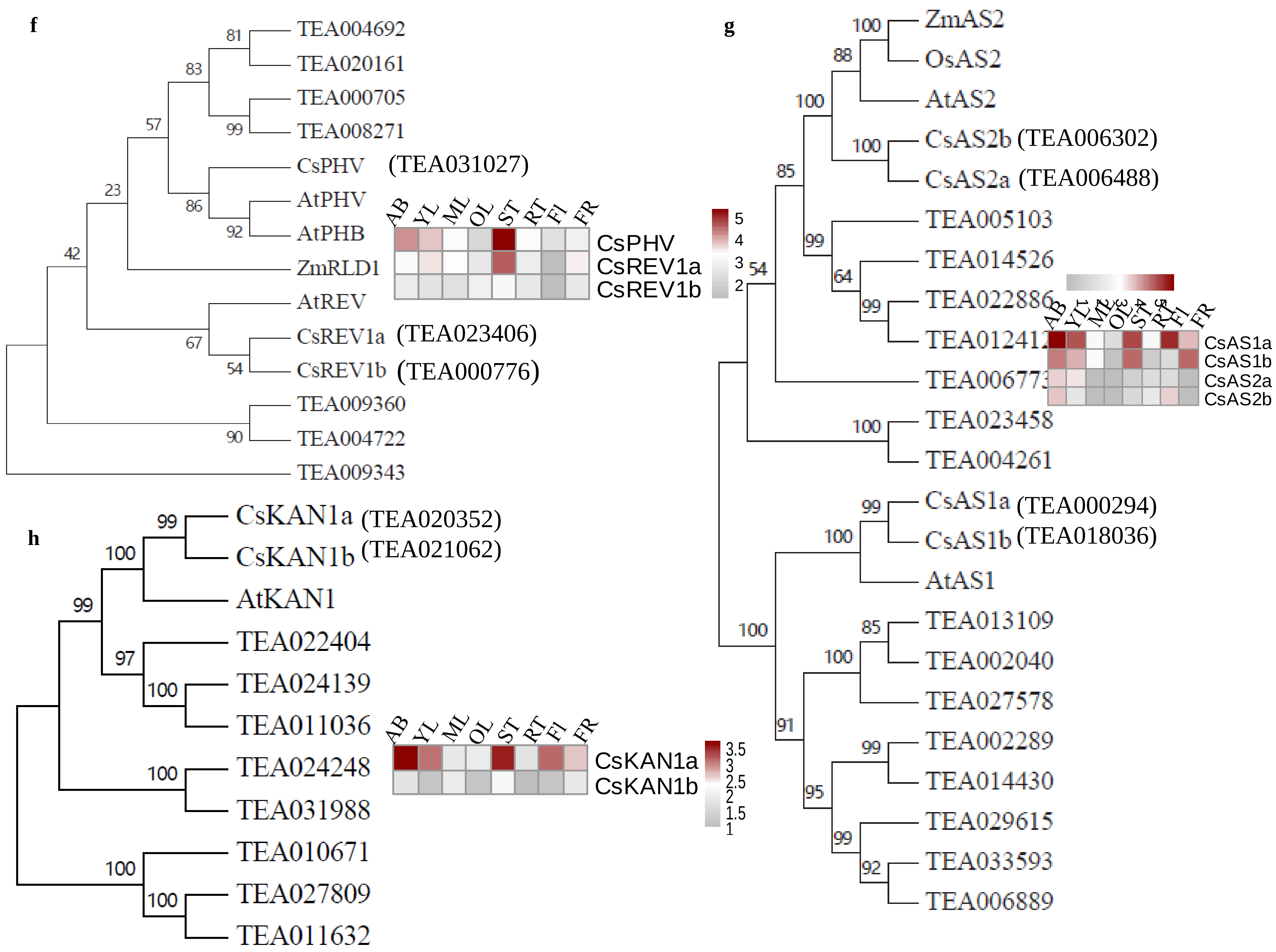

f
81
83
57
99
86
92
23
42
67
54
90
TEA004692
TEA020161
TEA000705
TEA008271
CsPHV (TEA031027)
AtPHV
AtPHB
ZmRLD1
AtREV
CsREV1a (TEA023406)
CsREV1b (TEA000776)
TEA009360
TEA004722
TEA009343
AB YL ML OL ST RT Fl FR
CsPHV
CsREV1a
CsREV1b
5 4 3 2
g
ZmAS2
OsAS2
AtAS2
CsAS2b (TEA006302)
CsAS2a (TEA006488)
TEA005103
TEA014526
TEA022886
TEA012412
TEA006773
TEA023458
TEA004261
CsAS1a (TEA000294)
CsAS1b (TEA018036)
AtAS1
TEA013109
TEA002040
TEA027578
TEA002289
TEA014430
TEA029615
TEA033593
TEA006889
100 88 100 100 85 99 64 99 54 100 99 100 100 85 100 91 99 95 99 92
1 2 3 4 5
AB YL ML OL ST RT Fl FR
CsAS1a
CsAS1b
CsAS2a
CsAS2b
h
CsKAN1a (TEA020352)
CsKAN1b (TEA021062)
AtKAN1
TEA022404
TEA024139
TEA011036
TEA024248
TEA031988
TEA010671
TEA027809
TEA011632
99 100 99 97 100 100 100 100
AB YL ML OL ST RT Fl FR
CsKAN1a
CsKAN1b
3.5 3 2.5 2 1.5 1

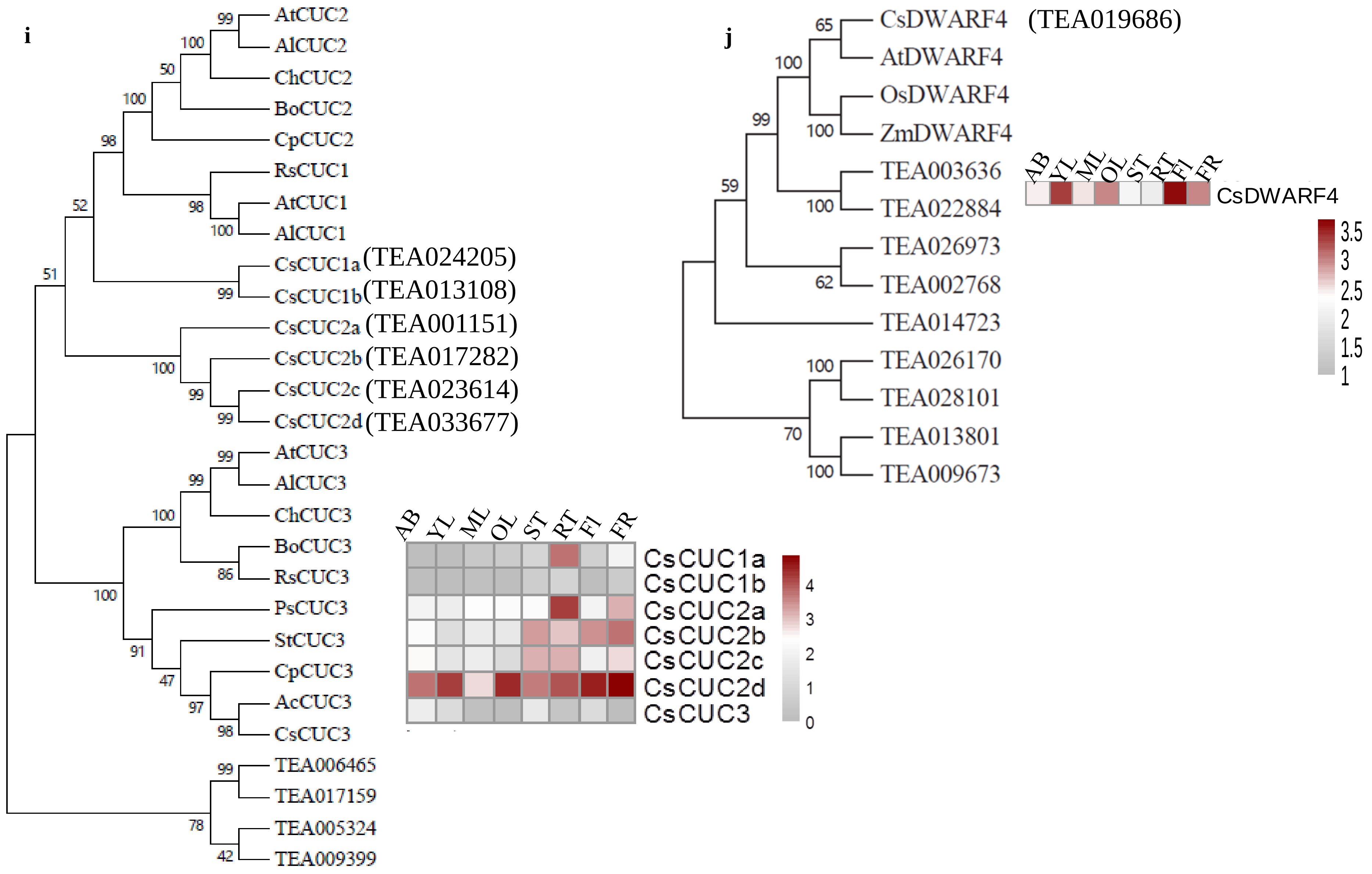

i
AtCUC2
AlCUC2
ChCUC2
BoCUC2
CpCUC2
RsCUC1
AtCUC1
AlCUC1
CsCUC1a (TEA024205)
CsCUC1b (TEA013108)
CsCUC2a (TEA001151)
CsCUC2b (TEA017282)
CsCUC2c (TEA023614)
CsCUC2d (TEA033677)
AtCUC3
AlCUC3
ChCUC3
BoCUC3
RsCUC3
PsCUC3
StCUC3
CpCUC3
AcCUC3
CsCUC3
TEA006465
TEA017159
TEA005324
TEA009399
AB YL ML OL ST RT FI FR
CsCUC1a
CsCUC1b
CsCUC2a
CsCUC2b
CsCUC2c
CsCUC2d
CsCUC3
j
CsDWARF4 (TEA019686)
AtDWARF4
OsDWARF4
ZmDWARF4
TEA003636
TEA022884
TEA026973
TEA002768
TEA014723
TEA026170
TEA028101
TEA013801
TEA009673
AB YL ML OL ST RT FI FR
CsDWARF4

k

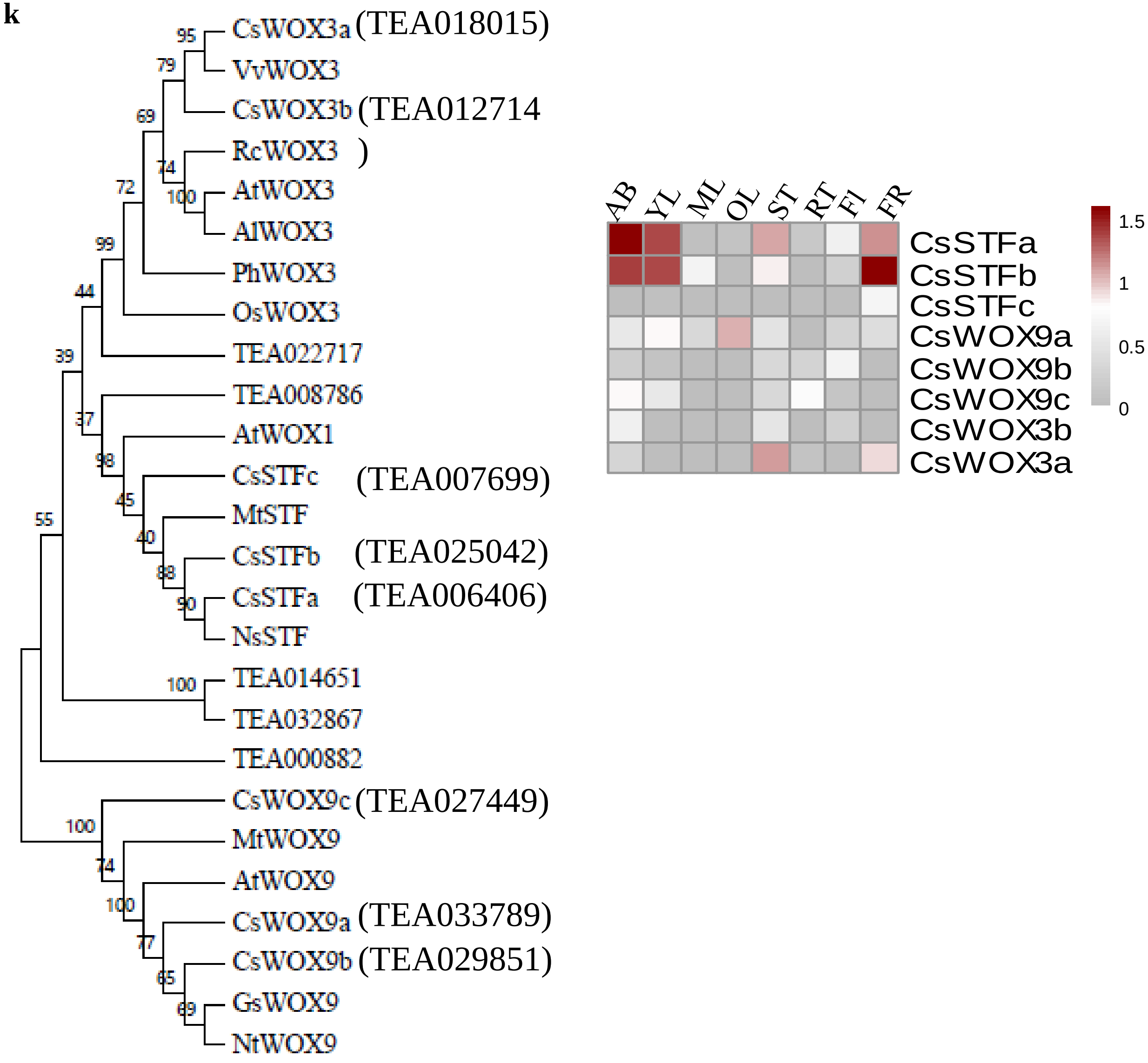

Supplemental Fig S6: The phylogenetic analysis and expression patterns of genes involved in *Camellia sinensis* leaf development.

The functionally characterized *Arabidopsis*, *Oryza sativa*, and *Zea mays* homologue proteins were used as references.
Amino acid sequences were aligned by using Clustal W, and MEGA 6.0 software was used to construct the phylogenetic tree by the NJ method with 1000 bootstrap replicates. Expression of genes in different tissues of Camellia sinensis plants were retrieved from.

Supplemental Fig S7:

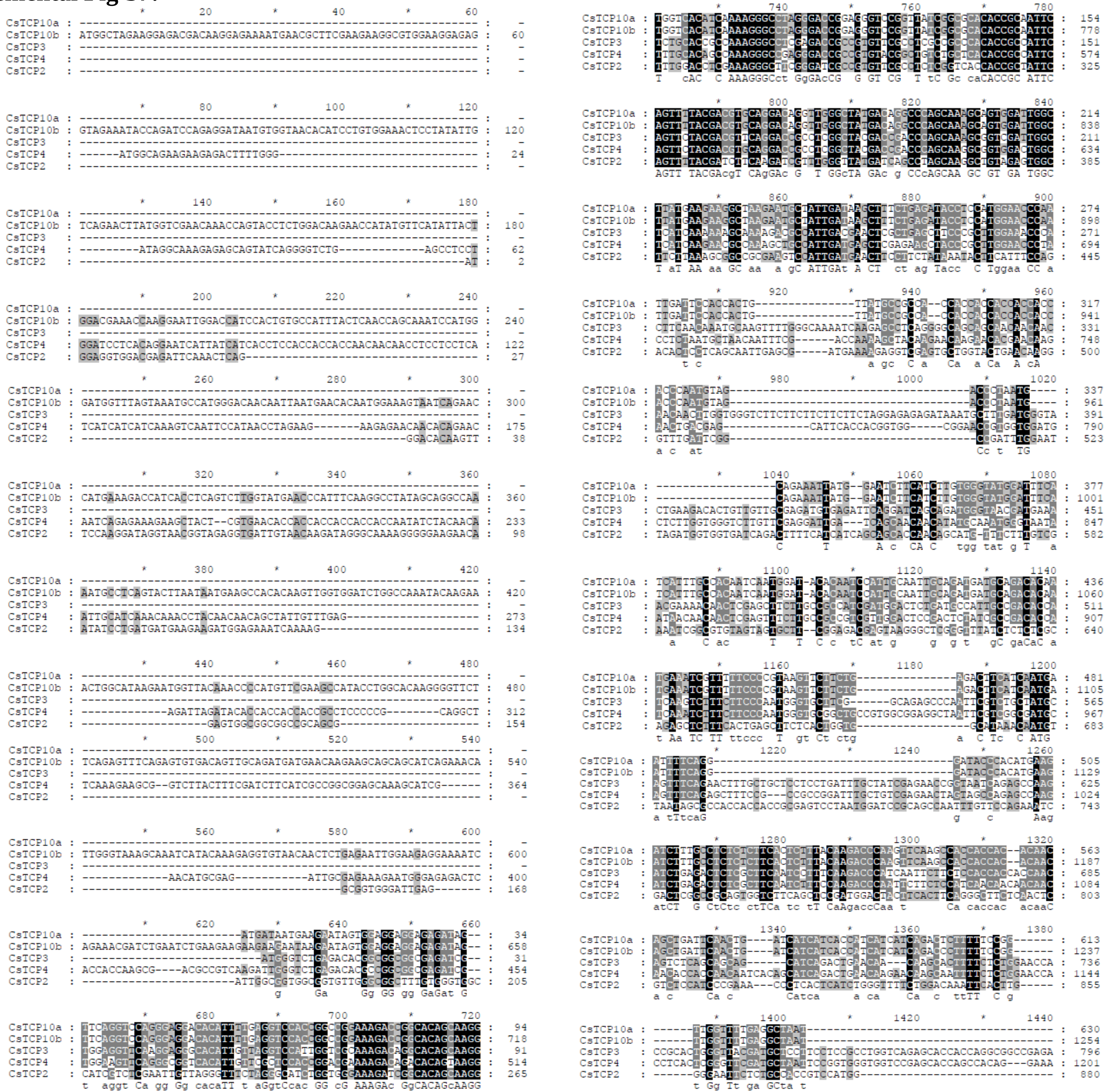

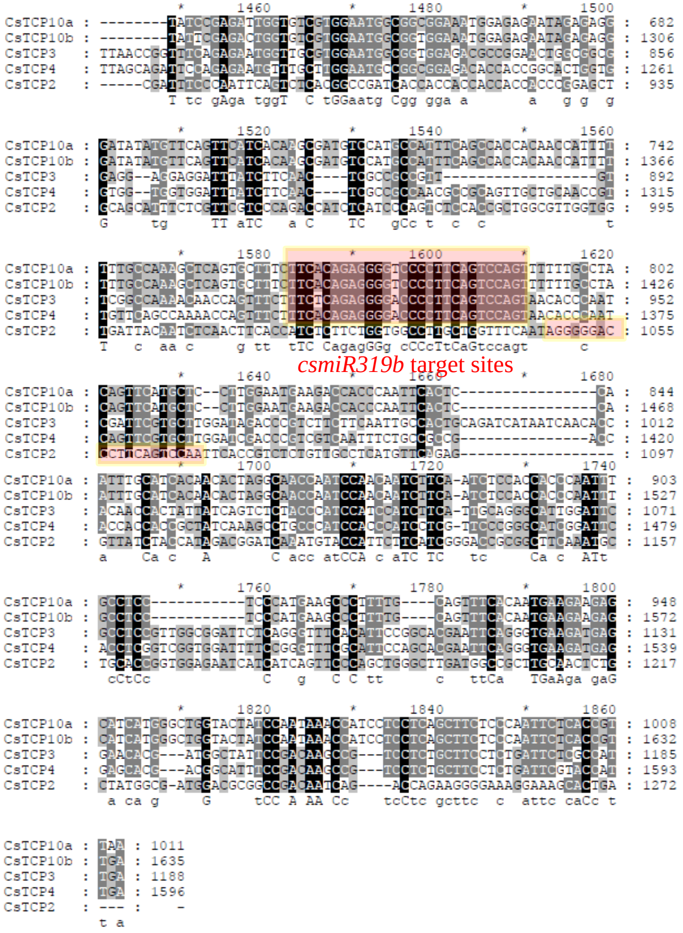

Supplemental Fig S7:Alignment of nucleic acid sequences of CIN-type Cs*TCP2,3,4* and *10* genes in *Camellia sinensis*.

Multiple sequence alignment was carried out using ClustalW and visualized with GenDoc software.

The *csmiR164b* target sites were highlighted in red frame color.

Supplemental Fig S8:

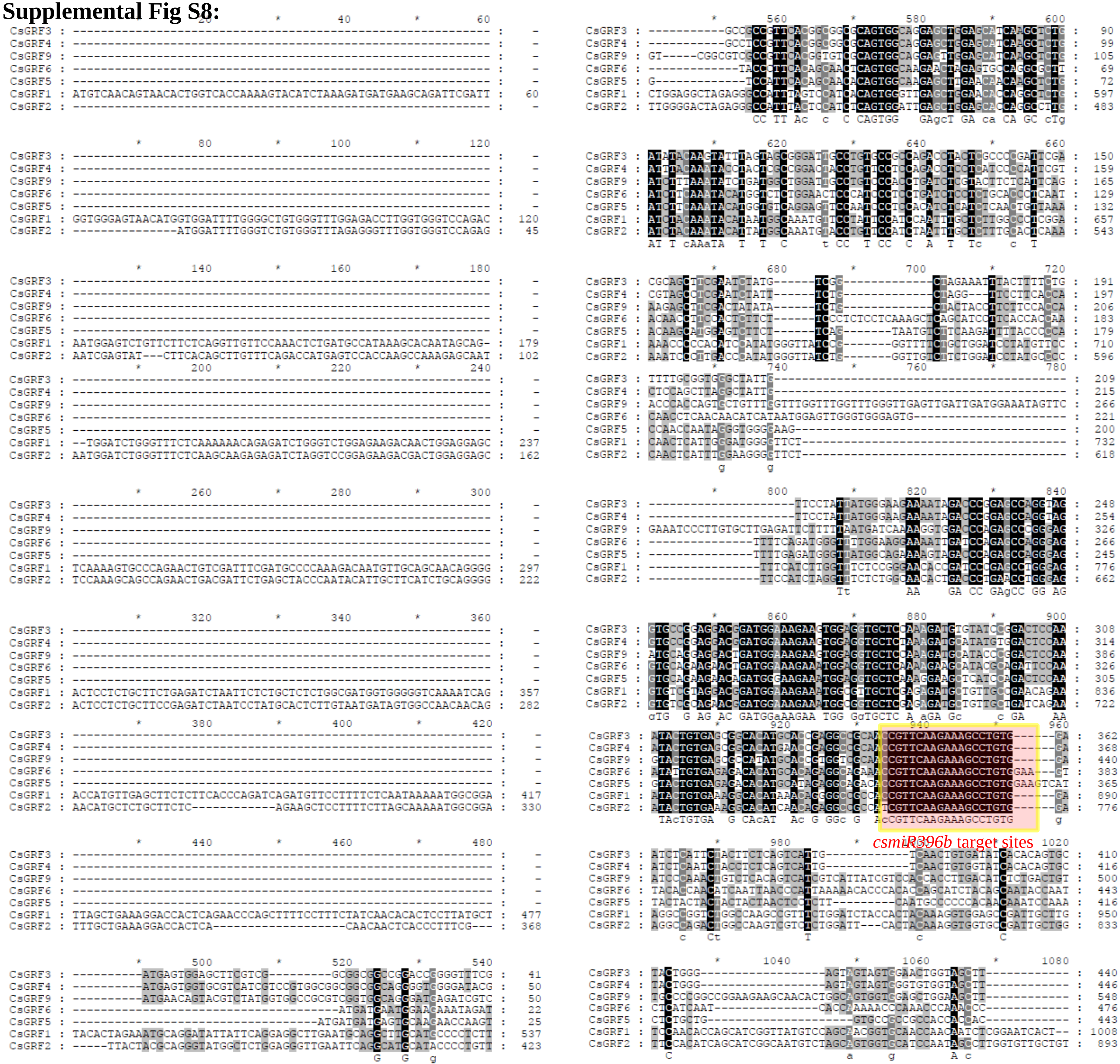

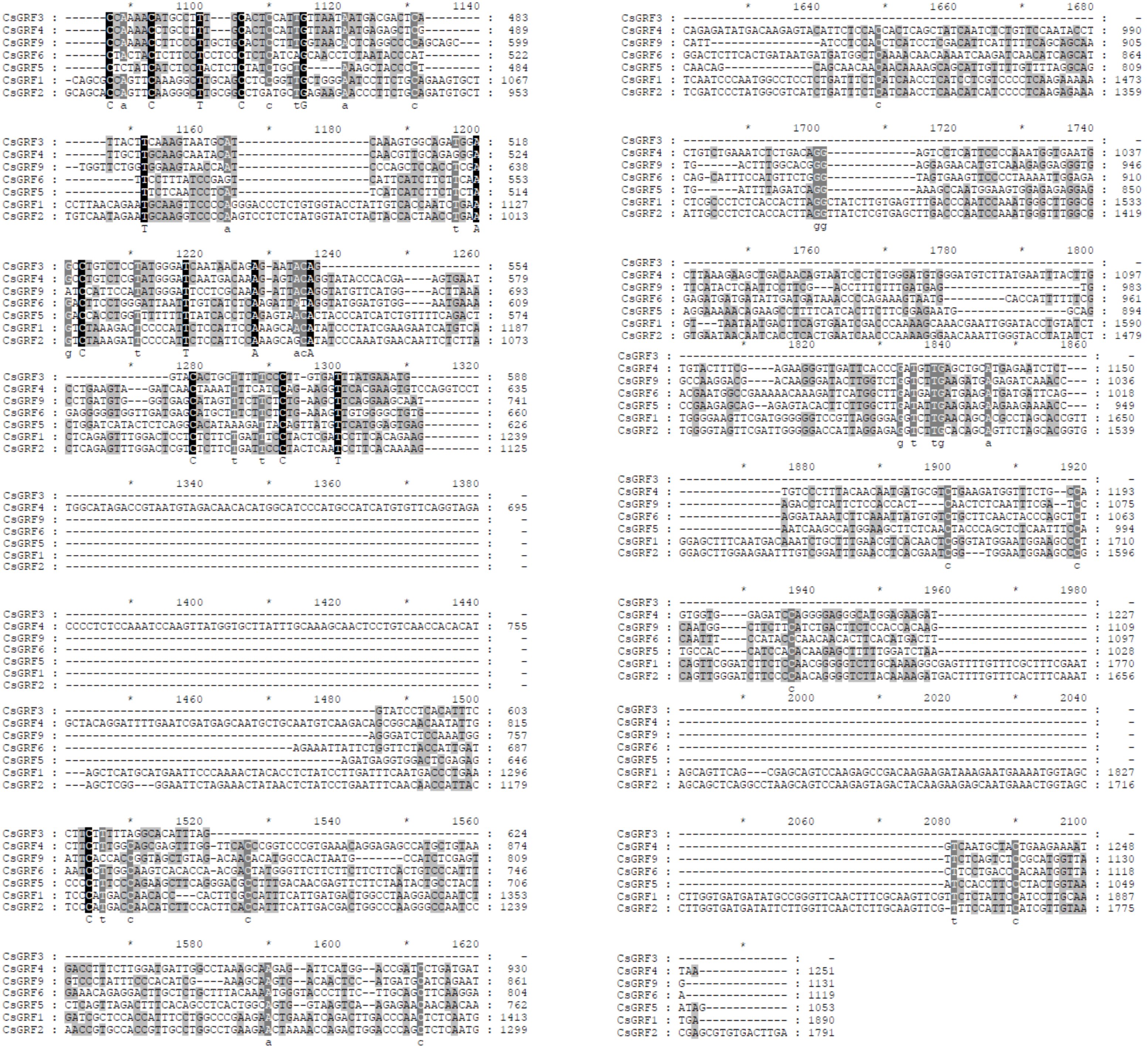

Supplemental Fig S8:Alignment of nucleic acid sequences of several *CsGRF* in *Camellia sinensis*.

Multiple sequence alignment was carried out using ClustalW and visualized with GenDoc software.

The *csmiR396b* target sites were highlighted in red frame color.

Supplemental Fig S9:

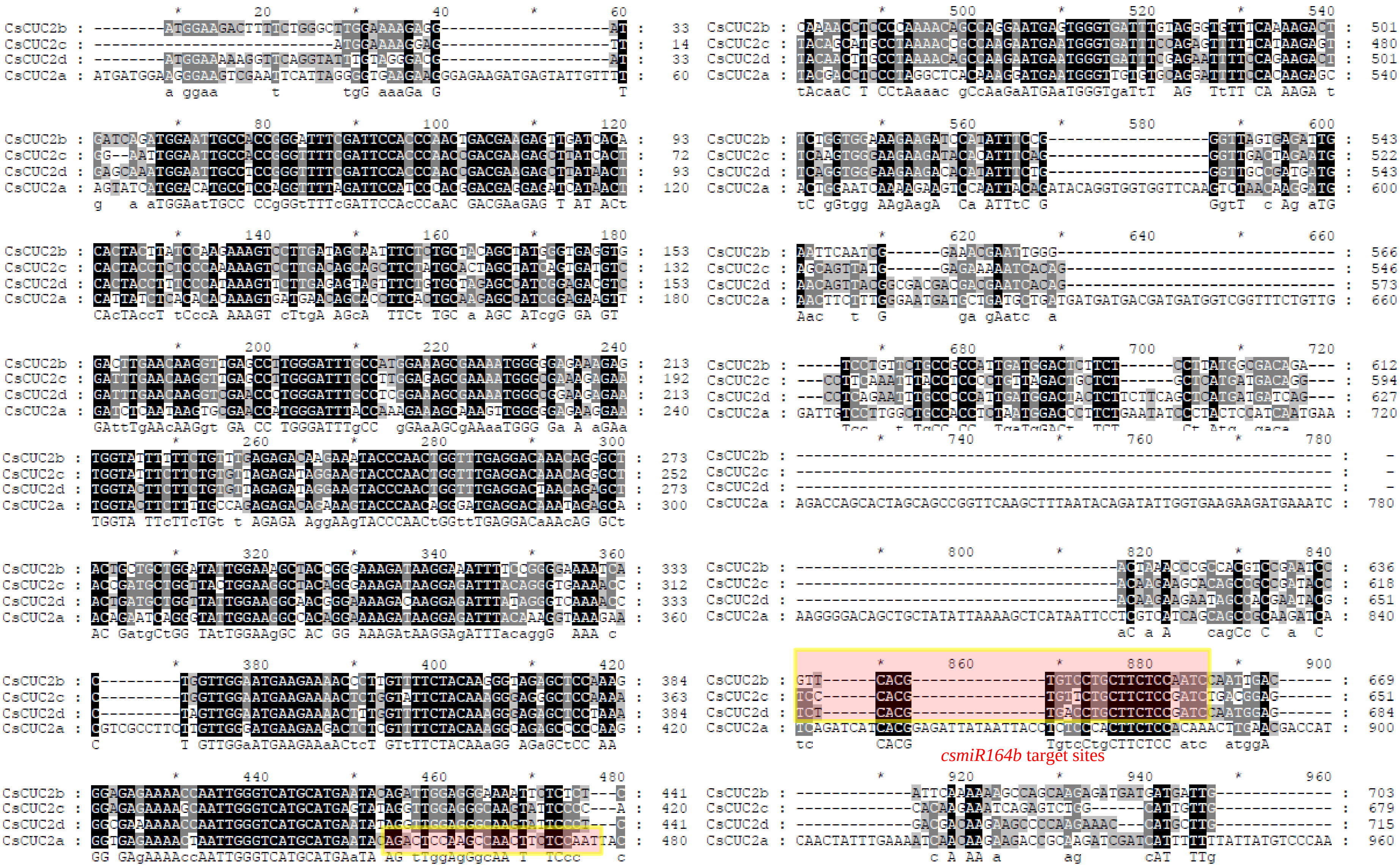

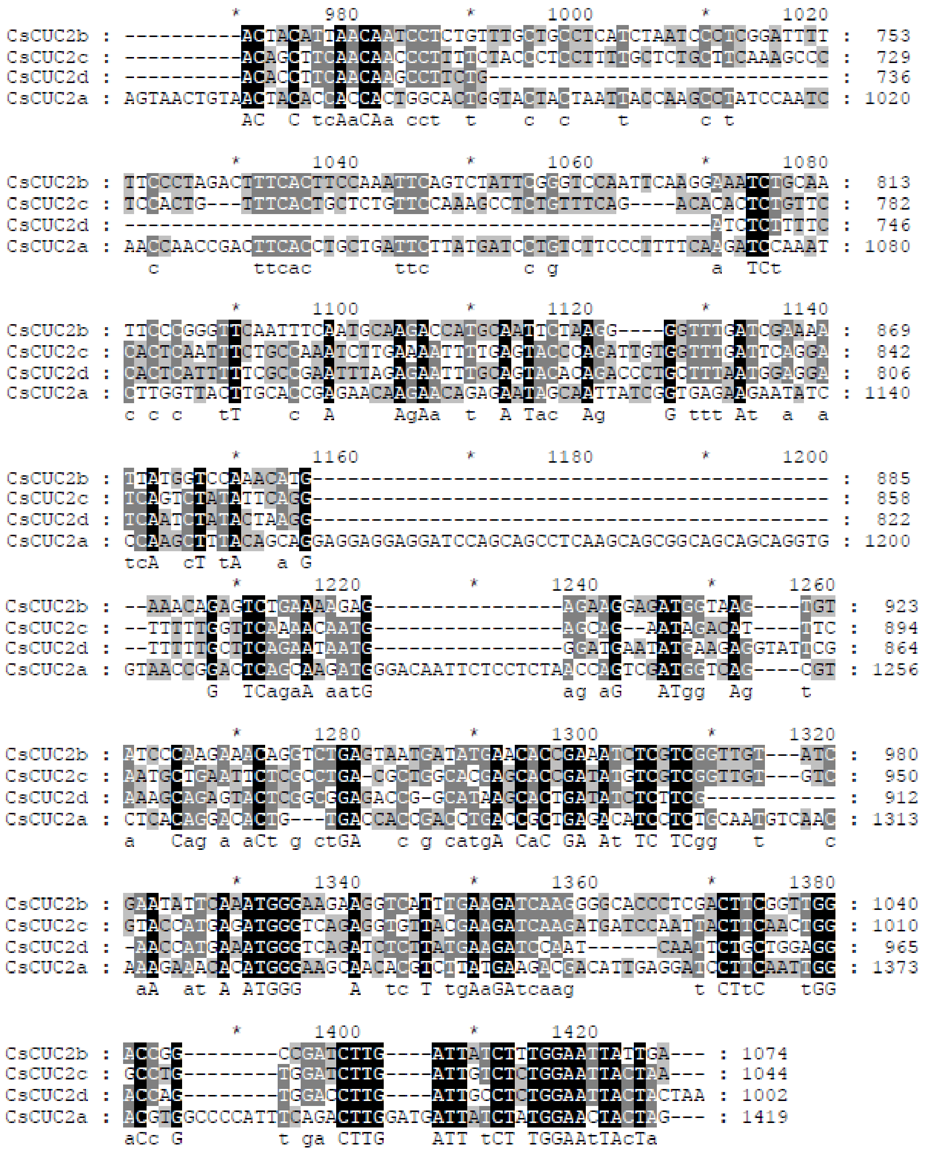

Supplemental Fig S9: Alignment of nucleic acid sequences of several *CsCUC2* genes in *Camellia sinensis*.

Multiple sequence alignment was carried out using ClustalW and visualized with GenDoc software.

The *csmiR164b* target sites were highlighted in red frame color.

Supplemental Fig S10:

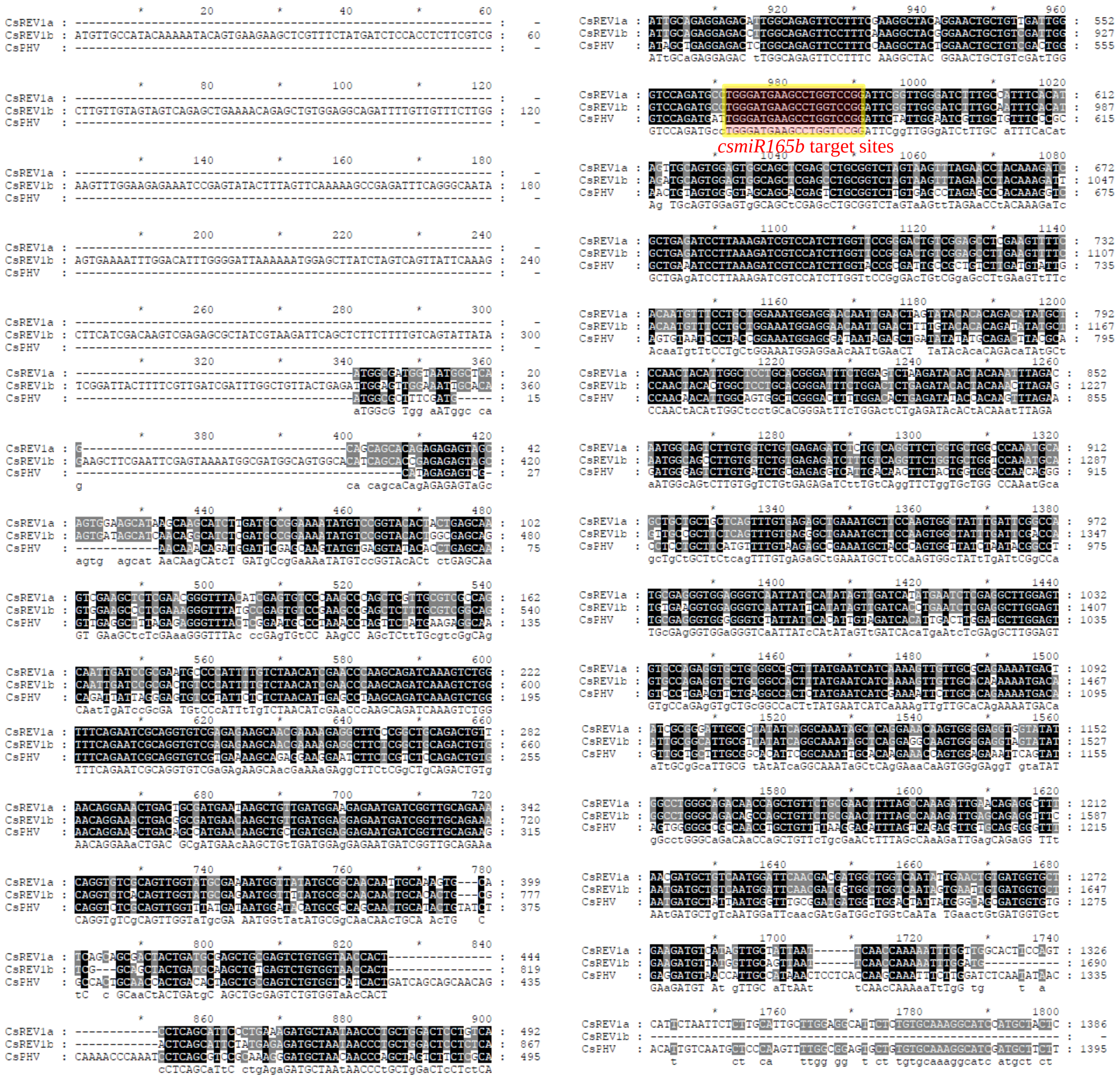

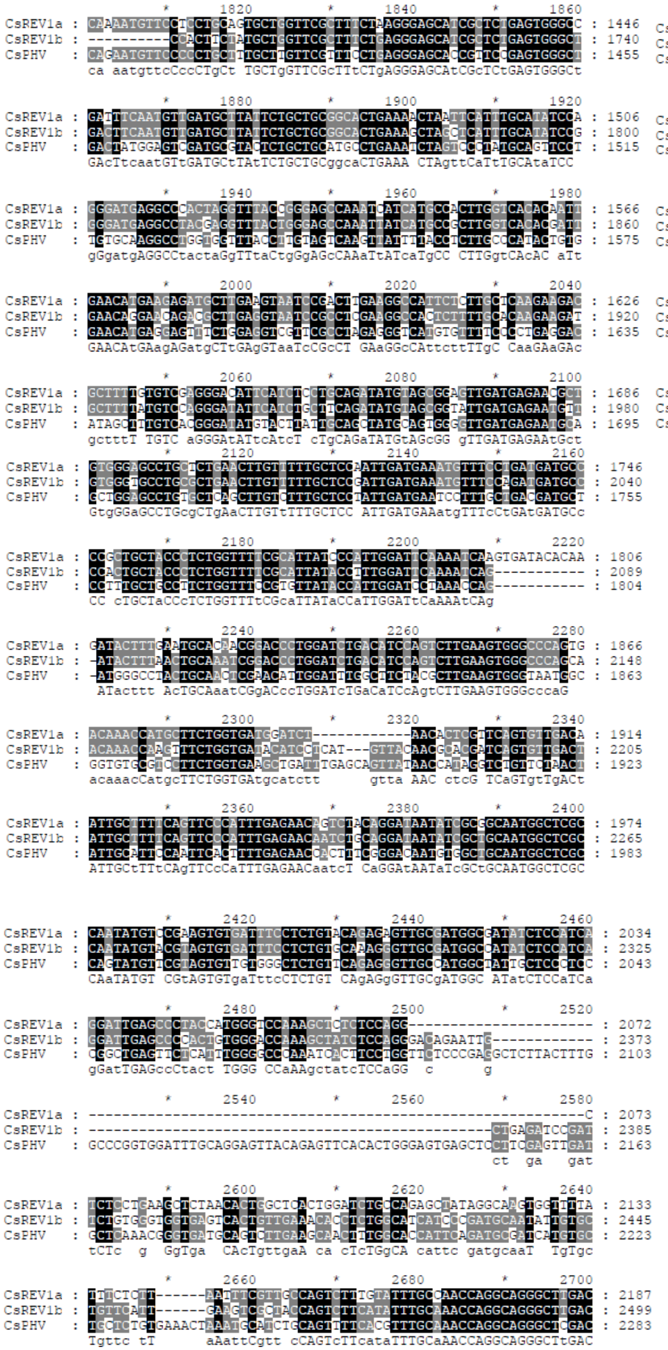

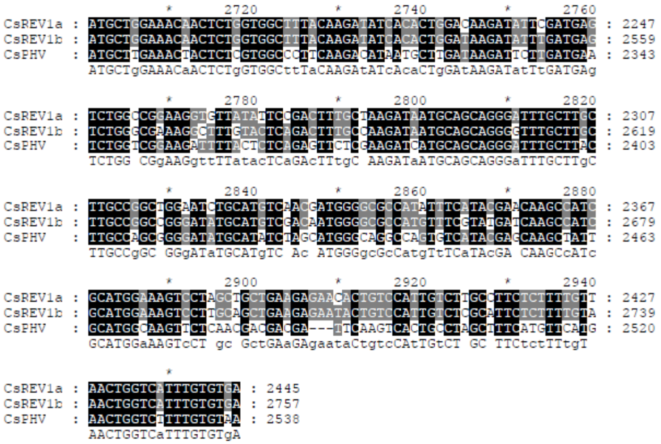

Supplemental Fig S10:Alignment of nucleic acid sequences of *CsREV1*and *CsPHV* genes in *Camellia sinensis* .

Multiple sequence alignment was carried out using ClustalW and visualized with GenDoc software.

The *csmiR165b* target sites were highlighted in red frame color.

Supplemental Fig S11:

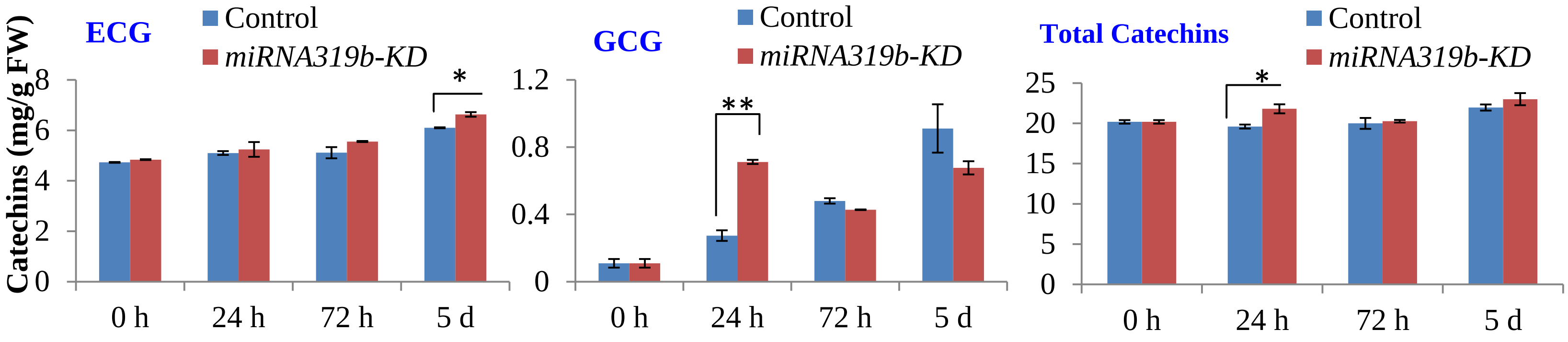

Supplemental Fig S11: Changes in catechins contents in csmiR319b-KD shoot tips with asOND.
